# Topological surface currents accessed through reversible hydrogenation of the three-dimensional bulk

**DOI:** 10.1038/s41467-022-29957-3

**Published:** 2022-04-28

**Authors:** Haiming Deng, Lukas Zhao, Kyungwha Park, Jiaqiang Yan, Kamil Sobczak, Ayesha Lakra, Entela Buzi, Lia Krusin-Elbaum

**Affiliations:** 1 Department of Physics, The City College of New York - CUNY, New York, NY 10031 United States; 2 Department of Physics, Virginia Tech, Blacksburg, VA 24061 United States; 3 Materials Science and Technology Division, Oak Ridge National Laboratory, Oak Ridge, TN 37831 United States; 4 Faculty of Chemistry, Biological and Chemical Research Center, University of Warsaw, 02-089 Warsaw, Poland; 5 City University of New York Graduate Center, New York, NY 10016 United States

**Keywords:** Electronic devices, Topological insulators

## Abstract

Hydrogen, the smallest and most abundant element in nature, can be efficiently incorporated within a solid and drastically modify its electronic and structural state. In most semiconductors interstitial hydrogen binds to defects and is known to be amphoteric, namely it can act either as a donor (H^+^) or an acceptor (H^−^) of charge, nearly always counteracting the prevailing conductivity type. Here we demonstrate that hydrogenation resolves an outstanding challenge in chalcogenide classes of three-dimensional (3D) topological insulators and magnets — the control of intrinsic bulk conduction that denies access to quantum surface transport, imposing severe thickness limits on the bulk. With electrons donated by a reversible binding of H^+^ ions to Te(Se) chalcogens, carrier densities are reduced by over 10^20^cm^−3^, allowing tuning the Fermi level into the bulk bandgap to enter surface/edge current channels without altering carrier mobility or the bandstructure. The hydrogen-tuned topological nanostructures are stable at room temperature and tunable disregarding bulk size, opening a breadth of device platforms for harnessing emergent topological states.

## Abstract (web summary)

Hydrogen can be incorporated within a solid and drastically modify its electronic and structural state. Here, the authors report reversible binding of H^+^ ions to chalcogens in the Bi_2_Te_3_ class of topological insulators and magnets, allowing Fermi level tuning into the bulk gap without altering carrier mobility or the bandstructure.

## Introduction

The ability to control carrier density—a key parameter of the electronic state of condensed matter—is central to achieving access to the topologically protected surface states in three-dimensional (3D) topological materials (TIs) that ideally are insulating in the bulk^1^. In the absence of magnetic dopants, the 2D electronic surface states with Dirac-type (linear) energy-momentum dispersion^1–3^ are gapless and fully spin-polarized, with protection against backscattering by local disorder guaranteed by time-reversal symmetry. These important layered van der Waals (vdW) quantum materials have narrow (≲300 meV) bulk gaps and charge carriers donated by intrinsic lattice defects^4^. As a result, the conduction through the bulk and its intermixing with the surface channels is what largely denies direct access to the surface currents sought for the implementation in topological spintronics and fault-tolerant quantum computing^5^.

Recent realizations of a nontrivial quantum anomalous Hall (QAH) state, featuring dissipation-free chiral edge currents^6^, made it apparent that when long-range magnetism and band topology combine^7^ the problem of bulk conduction can be particularly acute. Magnetic dopants, by breaking time-reversal symmetry, gap out the Dirac surface channels^8^, and when the Fermi level is in the Dirac (mass) gap the QAH state is expected to emerge. However, in addition to magnetic moment, the dopants also donate charge. Indeed, QAH was first observed in heavily Cr-doped (Bi,Sb)_2_Te_3_ ultrathin films^9^ in which to minimize bulk conduction a non-stoichiometric alloying of the constituent elements was employed and the film thickness had to be precisely controlled. Moreover, doping disorder restricted QAH temperature to the mK range.

In a newly discovered important class of intrinsic topological magnets (ITM)^10^ the doping and alloying disorders are avoided because the magnetic dopants are arranged as an atomic layer in a layered vdW crystal structure. For example, the Mn-based ITM MnBi_2_Te_4_ consists of Te–Bi–Te–Mn–Te–Bi–Te septuple layers (SLs), separated by vdW gaps and coupled antiferromagnetically^11^. As a consequence, the anomalous Hall (near) quantization was observed at somewhat higher temperatures^12^. However, such materials show high bulk carrier densities (typically > 10^20^ cm^−3^)^13,14^, and observation of QAH required delicate tuning of charge density that was again only possible in ultrathin (3–5 nm) samples with odd number of SLs. In thicker (hundreds of nanometers) samples ferromagnetism and quantization could be achieved when SLs were separated by a topological spacer^15^ yet tuning to quantization of either ferromagnetic (FM) or antiferromagnetic (AFM) ITMs without imposing severe thickness constraints is a major obstacle to realizing novel topological states.

Here we demonstrate a remarkably efficient and facile way to reversibly tune bulk carrier densities by over ≃ 10^20^ cm^−3^ in different classes of layered TIs^4^ by using insertion and extraction of ionic hydrogen to achieve access to topological surface states. The source of H^+^ ions is a dilute aqueous hydrochloric acid (HCl) solution, which leaves the TI crystal structure as well as electronic bands intact and has an extra benefit of removing native surface oxide while passivating surfaces and preventing reoxidation under exposure to air. We show that H^+^ in TIs, by preferentially forming an H–Te(Se) moiety within vdW gaps, donates electrons to the system and moves Fermi level *E*_*F*_ from the bulk valence (BVB) to bulk conduction (BCB) bands, crossing the bulk bandgap to display an ambipolar *p*- to *n-*type conductance conversion at the charge neutrality point. The process is fully reversible, as hydrogen-chalcogen moiety can be disassociated by a low-temperature annealing protocol under which hydrogen is easily removed. It is also multiply-cyclable and reproducible, thereby resolving one of the key limitations of magnetic and nonmagnetic TIs. In contrast with charge compensation by doping or alloying during growth^14,16^, the post-growth hydrogenation can tune *E*_*F*_ to charge neutrality without altering the band structure and can be effective in single crystals and in nano-devices, where the evolution of resistance under hydrogenation is easily monitored over time.

## Results

### Reaching charge neutrality and beyond in Bi_2_Te_3_ by hydrogenation

**Fig. 1:**
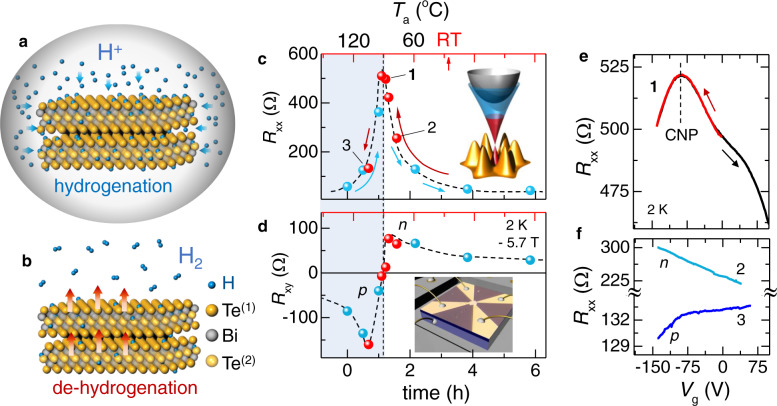
Tuning bulk conductivity of a topological material by hydrogenation. **a**, **b** Illustration of hydrogenation and de-hydrogenation process. **a** Hydrogenation: sample is submerged in a dilute aqueous HCl solution (0.5 M) at room temperature where H^+^ permeates the bulk through a timed diffusion process. **b** De-hydrogenation: H^+^ inside the sample is controllably released as H_2_ gas by an anneal in the 70–100 °C temperature range prescribed by the sample thickness, see Supplementary Note 1. **c** Longitudinal resistance *R*_*xx*_ and **d** Hall resistance *R*_*xy*_ at 2 K and −5.7 T of the initially *p-*type Bi_2_Te_3_ 121-nm-thick exfoliated device *vs*. hydrogenation time (bottom axis) show conversion (following blue arrows) in the same sample back to *n*-type (blue symbols). The back-conversion (red symbols) to *p*-type (following red arrows) is controlled by thermal annealing steps (30 min at each temperature, RT: room temperature, top red axis). Dashed lines are guides to the eye. *R*_*xx*_ increases by nearly two orders of magnitude near the charge-neutral point (CNP). The maximum is at the ambipolar point in *R*_*xy*_. Top inset: Bi_2_Te_3_ band structure rendering. Bottom inset: Optical image of the sample with van der Pauw contact geometry used. **e** Electrostatic backgating in the vicinity of the CNP (labeled **1**) reveals the expected maximum in *R*_*xx*_ at the CNP. The non-hysteretic *R*_*xx*_ vs. gating voltage *V*_g_ indicates an absence of surface traps. **f** Far from the CNP on the *n*-type (labeled **2**) or *p*-type side (labeled **3**), the *R*_*xx*_ maximum is inaccessible by voltage gating.

The ultralight hydrogen diffuses rapidly and easily incorporates into many materials^17^, although it shows qualitatively different behavior in different hosts^18^. Widely practiced in technology to passivate Si dangling bonds or enhance catalytic activity^19^, hydrogenation is also known to modify both electronic and structural states^20^ in a variety of diverse materials systems^21,22^—it was found effective in turning graphene into graphane^23^, inducing new magnetic phases in complex perovskites^24^, and modulating insulator-to-metal transition in a correlated Mott oxide VO_2_^25^. To ascertain the effects of hydrogen uptake on charge transport in the TIs we first chose well-characterized Bi_2_Te_3_ crystals with the conductivity flavor initially of a net acceptor type. The level of hydrogenation was controlled by timing the diffusion of H^+^ ions from a dilute HCl solution kept at room temperature (see Fig. 1a, b and “Methods”).

Our key and a most immediately notable result shown in Fig. 1 is a nearly two orders of magnitude increase in the low-temperature longitudinal resistance *R*_*xx*_ as a function of HCl exposure time to a maximum ${R}_{xx}^{\max }$ and a subsequent decrease as the exposure time is prolonged (Fig. 1c). The resistance maximum ${R}_{xx}^{\max }$ (conductance minimum) is at the charge-neutral point (CNP) where conduction is converted from *p-* to *n-*type, as determined from the corresponding Hall resistance (Fig. 1d). We surmise then that hydrogen, through the process of in-diffusion into the bulk (Supplementary Note 1), indeed donates electrons. This process is not self-limiting: at long (≥24 h) exposure times a decrease in *R*_*xx*_ on the deep *n*-side slows down yet continues. The observed ambipolar conduction, with well-distinguished *p* (hole) and *n* (electron) conduction regions, can be reproducibly reversed when hydrogen is removed by a low-temperature anneal (sketch in Fig. 1b) in the easily accessible range that depends on sample thickness. For the ~121-nm-thick Bi_2_Te_3_ crystal shown here the de-hydrogenation and a return from *n-*type across the CNP back to *p-*type is obtained within ~100 °C. Importantly, the carrier mobility during the entire process is not affected—e.g. in the sample in Fig. 1 it remains at ≅7000 cm^2^ V^−1^ s^−1^ (Supplementary Table 1).

After each annealing step, the system is room temperature stable and can be further finetuned by the conventional electrostatic gating. Figure 1e shows that once the carrier density is sufficiently reduced by H^+^, the vicinity of the CNP can be reproducibly explored by applying a gate voltage *V*_g_. *R*_xx_(*V*_g_) is precisely reversible upon changing the direction of the *V*_g_ sweep—the absence of hysteresis is a clear indication that hydrogenation does not create surface traps^26^ and is consistent with hydrogen permeating the bulk. We emphasize that in thick (≥100 nm) materials, far away from the CNP where carrier densities are in the high ~10^19^ cm^−3^ range, the CNP can not be reached by gating within a practically accessible voltage sweep (Fig. 1f).

### Salient features of 2D transport in Bi_2_Te_3_ under hydrogen

**Fig. 2:**
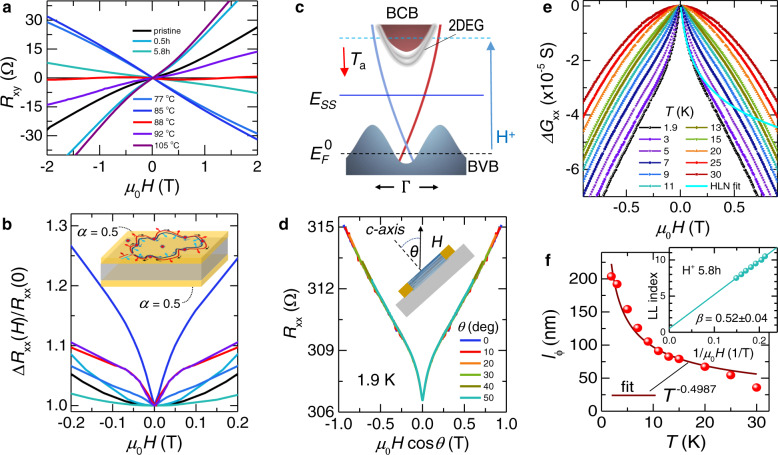
Charge transport across charge neutrality point. **a** Hall resistance *R*_*xy*_ vs. magnetic field *H* after hydrogenation and on annealing at different temperatures *T*_*a*_. Initially, the pristine Bi_2_Te_3_ crystal is *p*-type. The conversion from *p-*to *n-*type and back is indicated by the sign change of the slope *dR*_*xy*_/*dH*. **b** Evolution of magnetoresistance (normalized to the value at zero field) under annealing with time steps ${{\Delta }}t=30\,\min$ implemented to tune Bi_2_Te_3_ crystal to stable CNP; it evolves from a quadratic field dependence of a typical bulk metal to a weak antilocalization (WAL) regime with a characteristic low-field cusp near CNP. The cusp fit parameter *α* = 2 × 0.5 signifies contributions from top and bottom surfaces (0.5 from each), see text and Supplementary Fig. 1. **c** Band structure cartoon for Bi_2_Te_3_ illustrates the upshift of the Fermi level *E*_*F*_ on hydrogenation (cyan arrow) and the reversal by de-hydrogenation (red arrow). Bulk conduction and bulk valence bands are labeled BCB and BVB correspondingly. The 2D electron gas (2DEG) bands at the bottom of BCB are shown in gray, see details in the Supplementary Fig. 5. In Bi_2_Te_3_, the ${E}_{{{{{\rm{F}}}}}}^{0}$ at the CNP is slightly above the Dirac point (DP) and the Dirac electrons dominate the transport when *E*_*F*_ is within the bulk gap^1,2^. **d** Within the bulk gap, longitudinal sheet resistance *R*_*xx*_ vs. magnetic field scales with the out-of-plane field component ${H}_{\perp }=H\cos \theta$. **e** The change in WAL magnetoconductance Δ*G*_*xx*_ vs. magnetic field at different temperatures. With increasing temperature the WAL cusp in Δ*G*_*xx*_ smoothly vanishes and above ∼30 K transforms into a classical parabolic magnetoconductance. **f** The quantum dephasing length ${l}_{\phi }\propto 1/\sqrt{T}$, obtained from the fits to 2D localization theory (HLN^28^, see text), is characteristic of the 2D transport. Inset: Hydrogenation preserves the topological *π*-Berry phase estimated from SdH quantum oscillations (see Supplementary Fig. 6).

The type conversion on hydrogenation and de-hydrogenation is clearly seen in the magnetic field dependence of Hall resistance *R*_*xy*_ (Fig. 2a), with *R*_*xy*_ flipping its slope d*R*_*xy*_/d*H* and Hall coefficient ${R}_{{{{{\rm{H}}}}}}=-\frac{1}{{n}_{b}e}$ changing sign in the conversion region, where *n*_*b*_ is the carrier density obtained from the linear part of the high-field slope of *R*_*xy*_ (see Supplementary Fig. 1). As we approach the CNP, weak antilocalization (WAL) quantum correction to ‘classical’ conductivity at low magnetic fields emerges from a parabolic (∝*B*^2^) background as a *positive* magnetoresistance cusp (Fig. 2b), characteristic of a TI^27^. The number *n*_*Q*_ of quantum conduction channels contributing to WAL can be estimated from 2D localization theory^28^ ${{\Delta }}G(B)\simeq \alpha \frac{{e}^{2}}{2{\pi }^{2}\hslash }f(\frac{{B}_{\phi }}{B})$, where Δ*G*(*B*) is the low-field quantum correction to 2D magnetoconductance, coefficient *α* = *n*_*Q*_/2 equals to 1/2 for a single 2D channel, *f*(*x*) ≡ *lnx* − *ψ*(1/2 + *x*), *ψ* is the digamma function, and field ${B}_{\phi }=\frac{\hslash }{4e{l}_{\phi }^{2}}$ is related to the dephasing length *l*_*ϕ*_ of interfering electron paths. The fit to WAL conductance (see Fig. 2e and Supplementary Figs. 2 and 3) yields *α* ≃ 1.004 ± 0.005, corresponding to two 2D quantum channels we associate with top and bottom surfaces^27^. Figure 2c schematically depicts the Bi_2_Te_3_ band structure^2^ as the Fermi level *E*_F_ moves from BVB to BCB (hydrogen in) and in reverse (hydrogen out). The 2D character of WAL cusp when *E*_F_ is within the bulk gap is apparent in the scaling with the transverse component of magnetic field ${H}_{\perp }=H\cos \theta$ (Fig. 2d and Supplementary Fig. 2), and in the characteristic 2D temperature dependence of ${l}_{\phi }\propto 1/\sqrt{T}$ (Fig. 2f). The Drude-like *B*^2^ contribution to magnetoresistance is consistent with the anisotropy of bulk Bi_2_Te_3_ (Supplementary Fig. 4).

Near the CNP, where the net residual bulk carrier density is very low, *R*_*xy*_ displays a characteristic low-field ambipolar behavior (Supplementary Fig. 5a). The change in the net carrier density induced by hydrogenation is reflected in the Shubnikov-de Haas (SdH) quantum oscillations of Hall resistance at higher fields (Supplementary Fig. 5). An estimate of the Fermi surface size obtained from the change of the oscillation period in ∂Δ*R*_*xy*_/∂*μ*_0_*H* (Supplementary Fig. 5c, d) shows that the Fermi vector *k*_F_ is much reduced after hydrogenation—near the CNP *k*_F_ ≈ 0.014Å^−1^ and the corresponding surface carrier density *n*_2*D*_ ≅ 6.3 × 10^11^ cm^−2^ is very low (see Supplementary Table 2). We note that the 2DEG states arising from the subsurface band-bending of bulk channels present just below the BCB (Fig. 2c and Supplementary Fig. 5b) may also contribute to the 2D WAL. A conventional methodology we use to estimate Berry phase *φ*_*B*_ = 2*πβ* from the SdH oscillations^29^ yields Berry factor *β* ≅ 0.5 near the CNP and after 5.8 hours of exposure to hydrogen. Thus, the topological *π*-Berry phase^1^ (see inset in Fig. 2f and discussion following Supplementary Fig. 6) appears robust under hydrogen and not measurably affected by 2DEG.

### Preferential location of hydrogen in the van der Waals gaps

**Fig. 3:**
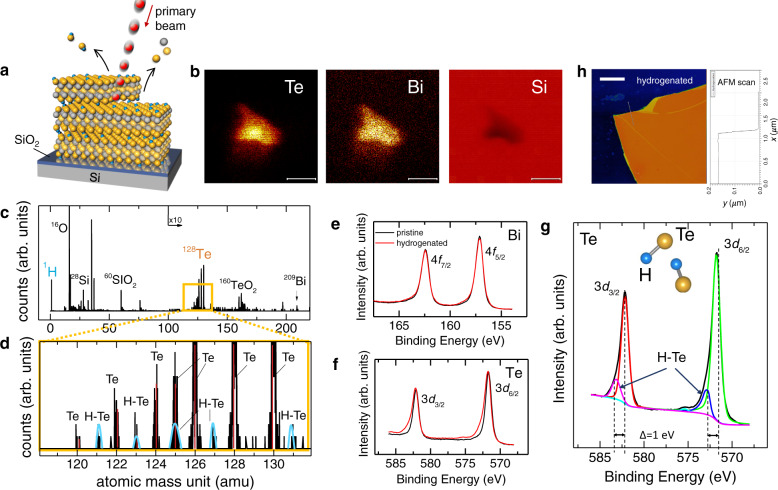
Spectroscopic detection of hydrogen in Bi_2_Te_3_. **a** Time-of-flight secondary ion mass spectrometry (ToF-SIMS) generates mass spectrum of the outermost 1.5–2.0 nm surface region by bombarding it with a primary ion (Bi^+^) beam. **b** ToF-SIMS images of Te (left), Bi (middle), and Si substrate (right) indicate the rastered area from which the spectra were obtained (see “Methods”). The scale bar is 10 μm. **c** Mass analysis of the secondary ions under negative bias shows the presence of ^1^H, ^128^Te, ^209^Bi, and ^129^[Te–H] moieties. Well separated peaks of the known long-lived Te isotopes with the intensity ratios occurring in nature (Supplementary Table 3) are detected. **d** The expanded view of the Te isotope atomic mass range clearly shows additional peaks (highlighted in blue) of lower intensity that perfectly and sequentially follow each Te^*α*^ peak by precisely Δamu = 1. Some hydrogen peaks appear to overlap with Te^*α*^. **e** XPS data of Bi 4*f*_7/2_ and 4*f*_5/2_ for pristine Bi_2_Te_3_ and H^+^ treated for *t* = 12 h show no change after hydrogenation. **f** Te 3*d*_3/2_ and 3*d*_6/2_ are modified by hydrogenation. **g** Analysis of the Te 3d XPS peaks clearly shows H binding to Te with a shift in binding energy of Δ = 1 eV. **h** AFM image of a 165 nm-thick Bi_2_Te_3_ crystal after hydrogenation. The scale bar is 2.5 μm. Surface scans show that surface morphology and the sample thickness remain intact after hydrogenation.

Next we ask, where does hydrogen go? Our transport experiments demonstrate that in Bi_2_Te_3_ hydrogen donates electrons and stabilizes in its positive charge state as H^+^. To optimize its Coulomb interaction with the host electrons, it will tend to locate in the regions of higher electron charge density, i.e. in the vicinity of a more electronegative Te. To chase its physical presence we first utilize time-of-flight secondary ion mass spectrometry (ToF-SIMS), focusing on Te (Fig. 3a–d and Supplementary Fig. 7). A negative bias mass spectrum of secondary ions (see Methods) ejected from Bi_2_Te_3_ shows a cluster of peaks in the 120–130 amu (atomic mass unit) range (Fig. 3c) that belong to several naturally occurring isotopes of Te, see Supplementary Table 3. A closer look at the Te isotope cluster (Fig. 3d) clearly shows additional peaks that differ from each Te isotope by exactly Δamu = 1, consistent with each isotope binding with one hydrogen.

On a microscopic scale, we confirm the affinity between H and Te by x-ray photoelectron spectroscopy (XPS), see “Methods”. XPS spectra near the Bi *4f* and Te *3d* core levels show that the Bi core levels^30^ are unperturbed by hydrogen (Fig. 3e), while the spectral shapes of Te *3d* peaks are quantifiably modified (Fig. 3f, g). The upshift Δ = 1 eV in binding energy due to [Te–H]^−^ moiety is easily distinguished from free hydrogen or surface oxidation, as seen, for example, during hydrogen electro-catalysis^31^, where Bi *4f* spectral shapes are upshifted by ~2 eV and both Bi_2_O_3_ and TeO_2_ peaks are present. By contrast, XPS shows that HCl cleanly removes surface oxide, Cl is entirely absent, and annealing restores the original state (Supplementary Fig. 8 and Table 4). Importantly, the surface morphology is preserved, with RMS roughness of ≈ 0.2 nm (Fig. 3h and Supplementary Fig. 9).

**Fig. 4:**
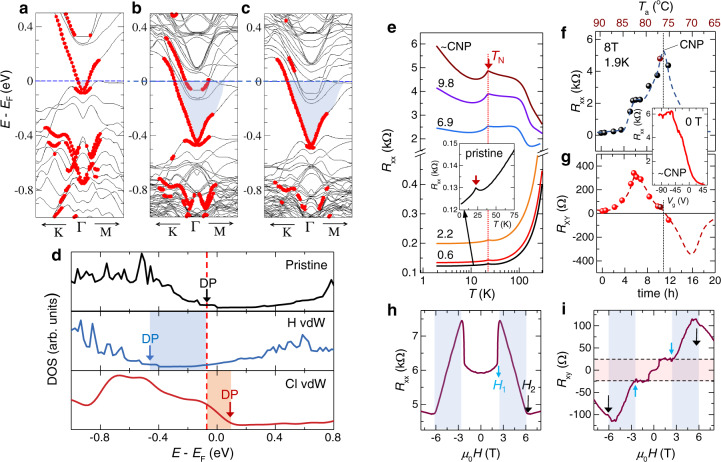
DFT-calculated band structures under hydrogenation and hydrogen-induced conductivity type conversion in a magnetic TI. DFT band structures for four quintuple layer (QL) slabs of **a** pristine Bi_2_Te_3_, **b** with interstitial hydrogen (H–Te^(2)^) bonding) within QLs and **c** with hydrogen in the vdW gap (H–Te^(1)^ bonding). The red color indicates the states localized at the top or bottom surface states. **d** Calculated total electronic densities of states (DOS). Top: For pristine Bi_2_Te_3_. Middle: With hydrogen in the vdW gaps (H vdW) acting as electron donor. Bottom: (Cl vdW) would act as electron acceptor, moving *E*_F_ in the opposite direction — the doping action not observed experimentally in transport and consistent with the absence of Cl in XPS. **e** Longitudinal resistance *R*_*xx*_(*T*) of a 80-nm-thick antiferromagnetic (AFM) MnBi_2−*x*_Sb_*x*_Te_4_ (*x* = 0.6) crystal on an expanded scale (see also Supplementary Fig. 19) clearly shows hydrogen-induced transformation from a metallic state in a pristine sample (inset) to an insulator-like bulk state at the CNP, with the low-*T* resistivity increased by nearly two orders of magnitude. The characteristic cusp (red arrow) occurs at the Néel temperature *T*_*N*_ ~ 25K, which is not affected by hydrogenation. **f** Longitudinal resistance *R*_*xx*_ and **g** Hall resistance *R*_*xy*_ at 1.9 K (below *T*_*N*_) and *H* = 0 as a function of hydrogenation time and annealing temperature *T*_*a*_. The maximum of *R*_*xx*_ is at the CNP where the conduction is converted from *p-* to *n-*type, and correspondingly the ambipolar behavior is observed in *R*_*xy*_. Dashed lines in **f** and **g** are guides to the eye. The hydrogenation data points are shown in black and red respectively, and annealing points in burgundy. Inset: Hydrogen-tuning close to the CNP enables finetuning *R*_*xx*_ by electrostatic backgating, with *R*_*xy*_ consistent with a zero-plateau expected in an AFM TI^32^. **h** *R*_*xx*_ and **i**, Hall resistance *R*_*xy*_ at 1.9 K measured at the CNP as a function of applied magnetic field. Below *T*_*N*_ and above the characteristic field *H*_1_ ≈ 3T (blue arrows) the system is driven into a canted AFM state, and becomes fully FM aligned at *H*_2_ ≈ 6T (black arrows). The surface Hall currents switch from a zero *R*_*xy*_ plateau to a finite plateau when some of the SLs align with the field.

The observed electron-donor action of hydrogen in a vdW TI through formation of hydrogen-chalcogen moiety is rationalized by the density functional theory (DFT) calculations (Supplementary Note 2 and Supplementary Figs. 10–12). The DFT band structures of Bi_2_Te_3_ (Fig. 4a–c) show that after hydrogenation the Dirac bands are preserved while the Fermi level *E*_*F*_ is upshifted into the BCB. This *n*-type doping is independent of whether H^+^ goes in interstitially or into the vdW gaps, however, the H–Te moiety appears most stable within the vdW gap (the formation energies are in line with XPS). The calculated local density of states (DOS) (Fig. 4d) highlights the shift of *E*_F_ relative to the Dirac point (DP). It also anticipates the opposite doping action of Cl, which in our experiments is not detected (Supplementary Table 4). Bi_2_Te_3_ structure maintains charge balance within each quintuple layer (QL) of atoms Te^(1)^–Bi–Te^(2)^–Bi–Te^(1)^, where the superscripts distinguish the inequivalent Te sites (see Supplementary Fig. 11a). The Te–Bi bonds are polar-covalent while only weak vdW type bonding exists between the neighboring Te^(1)^–Te^(1)^ planes with relatively weak and highly polarizable bonds. The hydrogen prefers going to the region between these planes, while the crystal structure remains intact (Supplementary Fig. 13).

## Discussion

The Fermi level tuning by hydrogenation demonstrated here is very general, indeed it is remarkably effective in other *p*-type chalcogen-based TI. We have achieved the type conversion across the CNP in Se-containing materials, such as Bi_2_Te_2_Se (Supplementary Fig. 14) and Ca-doped Bi_2_Se_3_ (Supplementary Figs. 15 and 16). Our DFT calculations confirm that a stable [H–Se]^−^ moiety similarly acts to move the Fermi level towards the conduction bands (Supplementary Figs. 11 and 12).

The technique is also very powerful in magnetic TI, and particularly in a very promising ITM class. One example is the intrinsic FM MnBi_2_Te_4_/Bi_2_Te_3_ superlattice^15^ where initial bulk carrier density is in the 10^20^ cm^−3^ range, yet type conversion across the CNP by hydrogen is obtained (Supplementary Figs. 17 and 18). Hydrogenation can also deliver an essentially continuous *E*_F_ tuning across the bulk gap of an intrinsic topological antiferromagnet, where the axion insulator behavior was very recently reported when the AFM ITM was made thin enough (~6 SLs or ~8 nm)^32^ to reduce the contribution from bulk conduction channels and to be able to deplete bulk carriers by electrostatic gating. Here we demonstrate in one exemplary AFM ITM that through hydrogen-tuning of *E*_F_ we can turn its bulk into an insulating state sans voltage gating. Figure 4e shows how the longitudinal resistance *R*_*xx*_(*T*) of a ten times thicker (80 nm) MnBi_2−*x*_Sb_*x*_Te_4_ (*x* = 0.6) crystal continuously transforms under hydrogenation from a metallic-like into a strongly insulating-like—a feat not yet achieved by a step-wise Bi–Sb alloying^13^. Under hydrogen uptake the Néel temperature *T*_*N*_ ~ 25*K* remains unchanged while the low-temperature *R*_*xx*_(*T*) increases by about two orders of magnitude, reaching maximum value at the CNP (Fig. 4e, f and Supplementary Fig. 19). Once near the CNP, *R*_*xx*_ can be finetuned by gate voltage *V*_*g*_ (inset in Fig. 4f, g and Supplementary Fig. 20). The Hall resistance *R*_*xy*_ (Fig. 4g) at the CNP displays ambipolar behavior, akin to the one in Bi_2_Te_3_ (Fig. 1d). The field dependencies of *R*_*xx*_(*H*) and *R*_*xy*_(*H*) under hydrogenation are consistent with the results previously only obtained in a few SL thin layers under voltage gating^12,32^, also see Supplementary Fig. 19. Near the CNP the magnetoresistance *R*_*xx*_(*H*) sharply changes at two characteristic fields: *H*_1_, above which the spin order is driven into a canted AFM state^13,14^, and *H*_2_ at which all spins align in a FM state (Fig. 4h). Below *H*_1_ Hall resistance *R*_*xy*_(*H*) exhibits a distinct plateau (where some of the SLs align with field) and a ‘zero-plateau’ (Fig. 4i) ubiquitous to AFM. Such plateaus — previously seen only in ultrathin exfoliated flakes^12,14^ where net magnetization *M* is set by the odd (FM) or even (AFM) number of SLs — are a manifestation of sensitivity of surface states to *M*. This is also consistent with the observed nearly-null *R*_*xy*_(*H* = 0) vs.*V*_g_, see Supplementary Fig. 20. Our key finding here is that by depleting free bulk carriers by hydrogenation we can access surface currents in bulk samples without modifying the band structure. This is in contrast to Bi-Sb alloying where the band structure is altered by addition of Sb and a topological transformation from an ITM to Weyl semimetal may occur^14^. And while the optimal ITM band structure for achieving high-temperature QAH is yet to be realized^33^, the hydrogenation technique provides a remarkably effective tool for tuning *E*_F_ into the Dirac gap.

Finally, we remark that a paucity of bulk-insulating TIs impedes the search for emergent quantum phenomena, with the prospect for real-world topological electronics still remaining far off. Hydrogen-tunability of high bulk carrier densities expands the availability of robust and easily accessible device platforms for harnessing topological phases with stunning macroscopic manifestations, such as dissipationless edge transport of charge, axion electrodynamics^34^ in topological magnets, and proximal topological superconductivity^1^ for quantum computing.

## Methods

### Crystal growth and structural characterization

The standard Bridgman-Stockbarger technique employing a vertical pull through the temperature gradient was used to grow single crystals of Bi_2_Te_3_, and Ca(0.09%)-doped Bi_2_Se_3_, and Bi_2_Te_3_Se^27^. X-ray diffraction of crystals was performed in a Panalytical diffractometer using Cu Ka (*λ* = 1.5405 Å) line from Philips high intensity ceramic sealed tube (3 kW) X-ray source with a Soller slit (0.04 rad) incident and diffracted beam optics. MnBi_2_Te_4_/Bi_2_Te_3_ crystals were grown by the vertical Bridgman method following the two-step technique^15^. In the first step, ground (20–100 mesh) high-purity (99.999%) bismuth (Bi), tellurium (Te) and manganese (Mn) were weighted according to the formula Mn_*x*_Bi_2−*x*_Te_3_ and loaded into the double-wall quartz ampules to avoid depressurization during the cooling process. The ampules were evacuated to 10^−6^ Torr. sealed, and loaded into a vertical furnace, and heated to 900 K, where they remained for 48 hr to achieve better homogenization. Afterwards, the furnace was cooled down to In the second step, the synthesized material was ground again, loaded into the ampules for the Bridgman growth, evacuated to 10^−6^ Torr, and sealed. To create the seed crystal, ampules with a small diameter (1.5–2.0 mm) along the tip in the lower end were used. Two ingots obtained from the first (synthesis) step were used to fill the Bridgman growth ampule. To obtain a homogenized solution, the material was heated to 1073 K and rotated along the ampule axis for 5 d in the hot part of the furnace. The samples were then moved down from the hot part at the speed of 2 mm/day. The temperature in the lower part of the furnace was kept at 873 K. This procedure resulted in n-type superlattice crystals with average sizes of 50-mm length and 14-mm diameter.

The samples for transmission electron microscopy (TEM) investigations (see Supplementary Figs. 12 and 13) were cut along the *c*-axis, in the [1120] orientation, using a focused ion beam (FIB) method. TEM characterization was carried out in a FEI Talos F200X microscope operated at 200 kV. Structural observations were performed in scanning transmission electron microscope (STEM) mode using a high-angle annular dark field (HAADF) imaging. Energy dispersive X-ray spectroscopy (EDX) using a Super-X system with four silicon drift detectors (SDDs) was applied to detection of differences in local chemical composition.

Crystals of MnBi_2−*x*_Sb_*x*_Te_4_ (*x* = 0.6) were grown out of a Bi(Sb)–Te flux^13,35^. Mixtures of Mn (Alfa Aesar, 99.99%), Bi and Sb pieces (Alfa Aesar, 99.999%), and Te shot (Alfa Aesar, 99.9999%) in the molar ratio of 1:10:16 (MnTe:Mn_2_Te_3_ = 1:5) were placed in a 2 ml alumina growth crucible and heated to 900 °C and held for 12 h. After slowly cooling across a ≈10 degree window below 600 °C in two weeks, the excess flux was removed by centrifugation above the melting temperature of (Bi,Sb)_2_Te_3_ (>585 °C). Crystals produced by this flux method were typically few mm on a side and often grew in thick, block-like forms with thicknesses up to 2 mm, but are easily delaminated.

### Hydrogenation and hydrogen detection

Hydrogenation of already-fabricated devices and single crystals was performed by submerging them in a dilute (0.5M) HCl + H_2_O = H^+^(H_2_O) + Cl^−^ solution at room temperature for a predetermined periods of time, then submerged and rinsed with deionized water and dried with N_2_. The diffusion of hydrogen H^+^ from the solution was controlled by the submerging time. Hydrogen was removed from within the samples by vacuum anneals in the 60–100 °C range. The rate of this process is prescribed by the annealing temperature and the thickness of the crystal—at higher temperature and/or for thinner crystals the diffusion rate of hydrogen to the surfaces is faster (see Supplementary Note 1).

### Time-of-flight secondary ion mass spectrometry (ToF-SIMS) and X-ray photoelectron spectroscopy (XPS)

The samples before and after hydrogenation were characterized with TOF-SIMS and XPS. Our hydrogenated samples are stable in air during the routine 30–40 min air exposure during sample handling during the process. In the TOF-SIMS sample preparation and loading steps, after hydrogenation samples were DI water rinsed, dried, but kept in air for 1–2 h before they were loaded into the vacuum chamber. The HCl does remove the oxide (as shown in Supplementary Fig. 8) but the surface may oxidize again after hours of exposure to air. The Physical Electronics nano-TOF TOF-SIMS was configured with a 20 kV Ar_2500_^+^ gas cluster ion gun for sputtering and 30 kV LMIG Bi^+^ analysis ion gun. In mass spectrum acquisition mode, the TOF-SIMS analysis ion gun was set in high-current bunched mode (Bi at 25 keV ion energy and 100 ns pulse duration). The TOF-SIMS data were acquired at a base pressure of ~10^−10^ Torr. XPS measurements were performed in Physical Electronics VersaProbe II XPS with a K*α* Al x-ray source (energy 1486.6 eV) and a hemispherical electron energy analyzer (pass energy 20 eV). The XPS energy-scale calibration was made with Bi 4*f*_*5/2*_ (162.5 eV), 4*f*_*7/2*_ (157.1 eV), Ag 3*d*_*5/2*_ (368.2 eV) and C 1*s* (284.8 eV) when available. PHI MutiPak software is used for XPS data reduction.

### Transport measurements

Transport measurements were performed in a 14 Tesla Quantum Design Physical property measurement system (PPMS) in 1 Torr (at low temperature) of He gas on many samples, each subjected to the same annealing protocol. The samples were annealed in situ starting from 330 K to 400 K. The annealing temperature ramp rate was 7 K per minute with the annealing time typically ~1 h. For *T*_*a*_ > 400 K, samples were annealed ex situ in a vacuum furnace. Crystals were mechanically exfoliated onto 300 nm SiO_2_/Si^+++^ wafers, typically resulting in micron-size crystals with thicknesses less than ~400nm, as determined by the atomic force microscope (AFM). Electrical contacts in the van der Pauw (vdP) configuration^36^ were photo-lithographically patterned and a sputtered Au metallurgy was used (Fig. 2c). Conformal Au coating amply covered side surfaces in order to make good contacts to top and bottom surfaces. The VdP dc measurements were carried out using a custom-configured electronic system in which four measurement configurations are switched by a Keithley scanner, with the current direction reversal employed for each measurement to minimize thermal *emf*. Sheet resistances in data analysis were calculated using the vdP formulae.

## Supplementary information

Supplementary Information

## Data Availability

The data that support the findings of this study are available from the corresponding author upon reasonable request.
