# Supplementary material for: Topological surface currents accessed through reversible hydrogenation of the three-dimensional bulk
Source: Nat Commun. 2022 Apr 28;13:2308. doi: 10.1038/s41467-022-29957-3 (PMC9050701; doi:10.1038/s41467-022-29957-3)
Supplement: Supplementary file 1 — Supplementary Information [file 41467_2022_29957_MOESM1_ESM.pdf]

Supplementary Materials:

Topological surface currents accessed through reversible hydrogenation of 3D topological materials

Haiming Deng[1], Lukas Zhao[1], Kyungwha Park[2], Jiaqiang Yan[3], Kamil Sobczak[4], Ayesha Lakra[1], Entela Buzi[1] & Lia Krusin-Elbaum[1,5]

[1]*Department of Physics, The City College of New York - CUNY, New York, New York 10031, USA*

[2]*Department of Physics, Virginia Tech, Blacksburg, Virginia 24061, USA*

[3]*Materials Science and Technology Division, Oak Ridge National Laboratory, Oak Ridge, Tennessee 37831, USA*

[4]*Faculty of Chemistry, University of Warsaw, 02-089 Warsaw, Poland and*

[5]*City University of New York Graduate Center, New York, New York 10016, USA*

The Supplementary Information File Contains Three Sections:

(I) SUPPLEMENTARY NOTES (2)

(II) SUPPLEMENTARY FIGURES (S1-S20) and TABLES (S1-S4)

(III) REFERENCES

I. SUPPLEMENTARY NOTES

Supplementary Note 1: Hydrogen Diffusion and Drift. Generally, the diffusion of hydrogen in semiconductors is complex since hydrogen can exist in several charge states [1] — as H^+ in *p*-type materials and as H^- and H^0 in *n*-type materials. In a solid, hydrogen can be present in atomic or molecular state, or bind to a defect or an impurity. The probability of formation of these different states depends on the defect type and concentration, and on hydrogen concentration. The apparent hydrogen diffusivity depends on the sample conductivity and on the hydrogen insertion method [2, 3]. Molecular formation and impurity trapping are known to retard hydrogen permeation in Si and GaAs and a similar behavior is expected in the chalcogen-based TIs.

The effective diffusion coefficient $D_{eff} = D_H\left[\frac{t_f}{t_f+t_B}\right]$ depends on the average time hydrogen spends in the free ($t_f \propto \varrho_f$) and bound ($t_B \propto \varrho_B$) states, where ϱ_f and ϱ_B are the corresponding hydrogen concentrations in the two states. The apparent diffusion coefficients is $D^* = D_{eff}\left(\frac{\varrho_B}{\varrho_f}\right)_0 exp\left[\frac{E_B-E_a}{kT}\right]$, where E_a is the activation energy for hydrogen diffusion and E_B is the binding energy of the hydrogen traps; D^* should decrease with trap concentration and trap binding energy. The diffusion profile in the simplest case can be described as $\varrho = \varrho_0 \, \mathrm{erfc}\left[\left(\frac{x^2}{4Dt}\right)^{1/2}\right]$, with the mean diffusion distance given by $x = (4Dt)^{1/2}$. Here $\mathrm{erfc}(z) = \frac{2}{\sqrt{\pi}}\int_0^z e^{-t^2}dt$ is the complementary Gauss error function. However, in real semiconductors (even in Si) the diffusion profiles rarely follow the simple formulas above. Since diffusing hydrogen is charged, the presence of electrostatic potential adds an additional term to the diffusion equation, and hydrogen ions can drift as a function of voltage bias [4, 5].

In our experiments, during hydrogenation, once all the H-Te bonds are saturated the diffusion reaches steady state. Our results on Bi_2Te_3 in Fig. 1c,d indicate that the HCl immersion time (bottom scale) and the annealing temperature (top scale) are surprisingly linearly correlated. This implies that the diffusion process is mainly governed by thermal activation. The correlation between hydrogenation and annealing times is illustrated for different annealing temperatures T_a and sample thicknesses in the figure to the right. At fixed T_a it follows $\propto (t-t_0)^p$ dependence, where $p = 0.37$ and t_0 is the time it takes to get to a given carrier density without annealing. We also note the reproducibility of R_{xx} and R_{xy} during in- and out-diffusion, and the consistency of R_{xx} at the CNP with electrostatic gating (Figs. 1c and 2e).

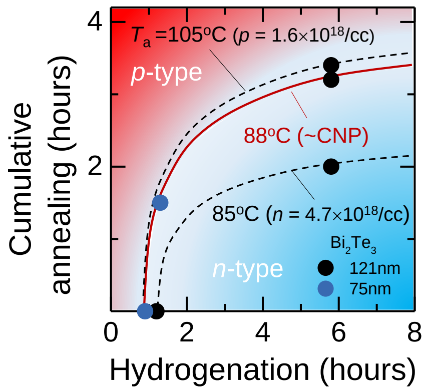

As in other semiconductors [5], we surmise that the annealing process can be described by

$$[Te^-H^+] + e^- \to Te^- + H \xrightarrow{\mathrm{H}} H_2, \tag{1}$$

and hydrogen is removed in the molecular form. The presence of Te-H moiety is confirmed by

our XPS and TOF-SIMS experiments (Fig. 3) and by the DFT calculations (Figs. 4 and S11a).

We remark that technologically the process is somewhat akin to hydrogen passivation of Si dangling bonds [6]. Hydrogenation is practiced at scale in the semiconductor industry, particularly in solar cell technology [7] as a way of improving cell efficiencies. With a potential for being enhanced by e.g. localized laser beams, as practiced in Si [8], the hydrogenation process in topological materials opens exciting opportunities for the charge-neutralization of advanced nano-architectures, including contact metallurgy and hetero-interfaces.

Supplementary Note 2: Density Functional Theory (DFT) Band Structure Calculations. We performed DFT calculations on a pristine Bi_2Te_3 slab and three Bi_2Te_3 slabs with hydrogen at interstitial sites or within the van der Waals gaps, using VASP [9, 10] code. The four slabs have thickness of four quintuple layers (QLs) which is about 4 nm. For the interstitial sites H-Te(2) bonding was considered, whereas for hydrogen within the van der Waals gaps H-Te(1) bonding and H-Te(1)-H bonding were separately considered. We henceforth refer to these three hydrogen-included structures as Case I, II, and III. For the pristine slab, we used a supercell of $1 \times 1 \times 4$QLs, while for the slabs with hydrogen we used a supercell of $3 \times 3 \times 4$QLs. For simplicity of the band structure, hydrogen was included in order to retain inversion symmetry. For Case I and II, two hydrogen atoms were included, while for Case III four hydrogen atoms were included in the supercells. We used Perdew-Burke-Ernzerhof (PBE) [11] generalized gradient approximation with projector-augmented-wave (PAW) pseudopotentials [12, 13]. Spin-orbit coupling was included self-consistently and van der Waals interaction was included using the DFT-D3 correction method of Grimme *et al.* [14].

The geometries of the slabs were fully relaxed until the residual forces were less than 0.01 eV/Å. The number of k points sampled is $11 \times 11 \times 1$ for the pristine slab, while it is $5 \times 5 \times 1$ for the hydrogen-included slabs, including the Γ point. In order to avoid interactions between neighboring supercells, a vacuum layer of about 20 Å was included in a supercell. After the geometry relaxation, in Case I, the H-Te(1) bond length is 1.74 Å, whereas in Case II and III, the H-Te(2) bond lengths are 1.91 and 1.92 Å, respectively. The formation energies of the defect for Case I, II, and III are -2.4, -2.6, and -1.8 eV per hydrogen, respectively. Since the formation energy of Case II is lowest, the most stable defect configuration is H-Te(1) bonding within the van der Waals gap. Figure S10 shows the calculated band structures of Case I, II, and III. The band structure of the pristine Bi_2Te_3 is shown in Fig. 4a in the main text.

We also performed DFT calculations on a pristine Bi_2Se_3 slab and a Bi_2Se_3 slab with hydrogen within the van der Waals gap (similar to Case II in the case of Bi_2Te_3) when the slabs are 4 QLs thick. We used the same parameter values and the same number of k points as in the case of Bi_2Te_3. The geometries of the two slabs were fully relaxed until the residual forces are less than 0.01 eV/Å. Considering the result of Bi_2Te_3 slab, we considered H-Se(1) bonding only. The

H-Se(1) bond length is 1.50 Å. The formation energy of the defect is −2.1 eV per hydrogen. Since the formation energy is negative, this defect is stable. Supplementary Figure 11 shows the calculated band structures of the pristine Bi_2Se_3 slab and the slab with Se(1)-H bonding. The H-Se(1) bonding moves the Fermi level E_F into the conduction band, similarly to the case of Bi_2Te_3 — compare Supplementary Figure 10b to 11c. Figure S12 shows calculated DOS for the pristine Bi_2Se_3 and the slab with H-Se(1) bonding. This shift of the E_F and the calculated DOS plots support the electron doping effect of hydrogen on the Bi_2Se_3 slab. To reduce high computational cost, we used thin slabs which produced a small gap at the Γ point caused by hybridization between top and bottom surface states. This gap is closed for thicker slabs. Note that the experimental samples are several hundreds nanometers thick. The electron doping effect of hydrogen would not change for thicker slabs. The size of the surface hybridization gap for Bi_2Se_3 differs from the corresponding gap size for Bi_2Te_3.

We note that both Te and Se dihydrides have been known to form. Te is isoelectronic to Se — it is the next group VI-A element. Its larger atomic core and weaker electronegativity than Se means that Te will exhibit a different chemistry. H_2Se [15] is stable as gas molecules and as a solid at ambient pressure, but H_2Te gas molecules are unstable and rapidly decompose into the constituent elements (above -2°C [16]).

II. SUPPLEMENTARY FIGURES AND TABLES

	Pristine	30min (H^+)	5.8h (H^+) & Seq-annealed (105°C)
R_{xx}(Ω)	58.82	125.7	133.2
Mobility (cm^2/V·s)	7141	7239	7138
n_{3D}^{Hall} (×10^{18} cm^{-3})	3.0	1.8	1.7
n_{3D}^{sdH} (×10^{18} cm^{-3})	0.912	0.603	0.554

Supplementary Table 1| Carrier mobility unaffected through the hydrogenation/dehydrogenation cycle. From left to right: The longitudinal sheet resistance and carrier mobility at 1.9 K for a pristine sample with p-type carriers, the sample after 30 minutes of hydrogenation (still p type), and through the hydrogenation and de-hydrogenated cycle (p to n then back to p). To compare carrier mobilities under different hydrogenation conditions the Fermi level was tuned to be roughly the same as indicated by the 'distance' of R_{xy} from the ambipolar point in each case, see Fig. 1d. Carrier mobility remains unchanged within 1.4%. Mobilities were extracted from R_{xx} using the Hall data at high magnetic fields in the linear regime (see Supplementary Figure 1 below) from which the 3D Hall carrier densities n_{3D} were obtained. 3D carrier densities obtained from SdH (Supplementary Figure 5 and Table 2) are shown for comparison. The factor of 3 difference arises from hexagonal warping distortion of the Fermi surface prominent in Bi_2Te_3 on the BVB side [17, 18].

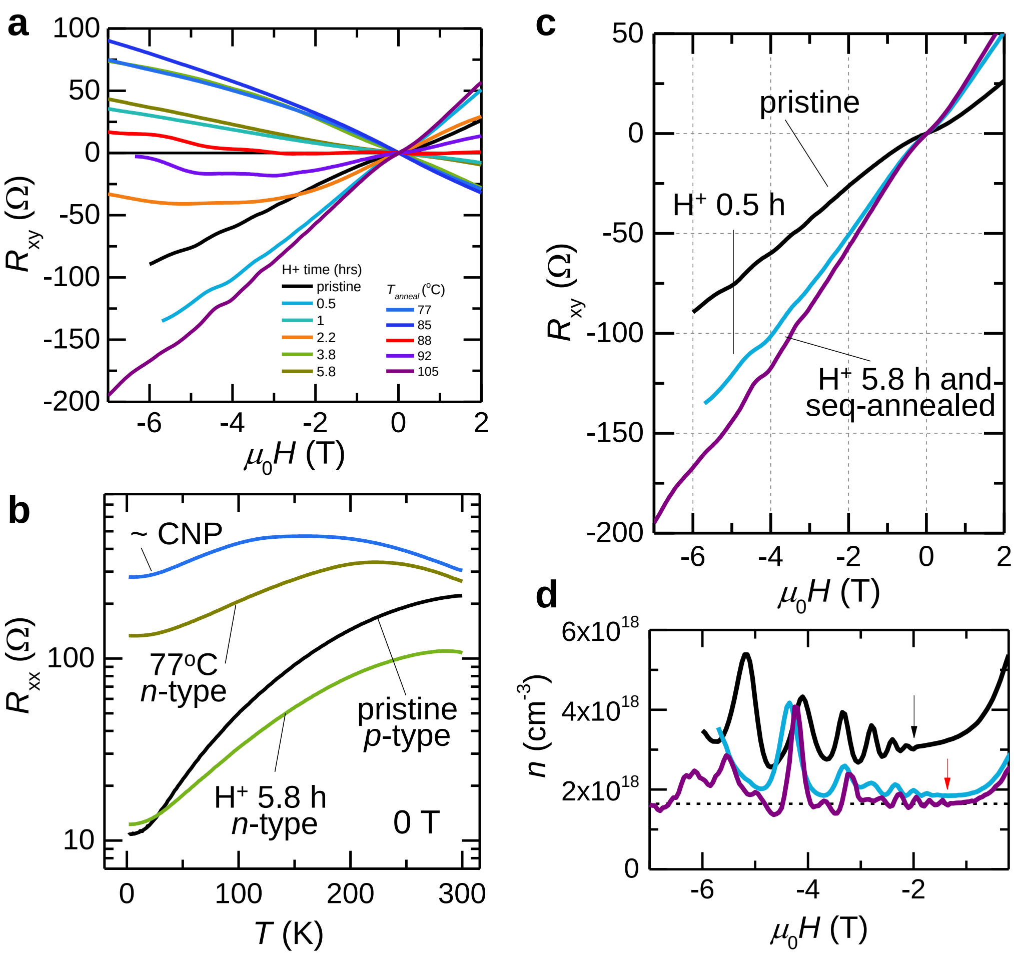

Supplementary Figure 1| High-field scans of Hall resistance R_{xy}. **a**, Hall resistance vs. magnetic field shown here on the hydrogenation cycle for different hydrogenation times and on the de-hydrogenation (annealing) cycle for different annealing temperatures. The observed nonlinearity in the ambipolar regime in the vicinity of the CNP is due to charge compensation, i.e. where the holes are compensated by electrons donated by hydrogen. **b**, Longitudinal resistance vs. temperature at zero magnetic field shows a clear evolution from a metallic-like in a p-type pristine sample, to the highest semiconducting-like near the CNP, where conduction type converts, continues to the n-type side and becomes metallic-like again (here shown after 5.8 h of HCl). Annealing reverses the process. **c**, The pristine Bi_2Te_3 crystals we use in our experiments are of extremely good quality, as they exhibit Shubnikov de Haas (SdH) oscillations already at a relatively low field of ~ 2 T and have a a relatively low bulk carrier density $n \cong 3 \times 10^{18}$ cm^{-3}. It is evident that the averaged $R_{xy}(H)$ is field-linear up to the highest field used in this study, and the slope ($\propto 1/n$) remains the same as in the 1-2 T range. **d**, The SdH oscillations are pronounced at the highest fields, which is reflected in the carrier density n obtained from the Hall constant $R_H = 1/ne$. In the ambipolar regime where both holes and electrons are present, and where the overall $R_{xy}(H)$ flattens in field and SdH oscillations have very long period (small Fermi surface, see Supplementary Figure 5), the determination of the net carrier density is not reliable, and in this regime the surface carrier density can be obtained from the SdH oscillations, see Table 2 below.

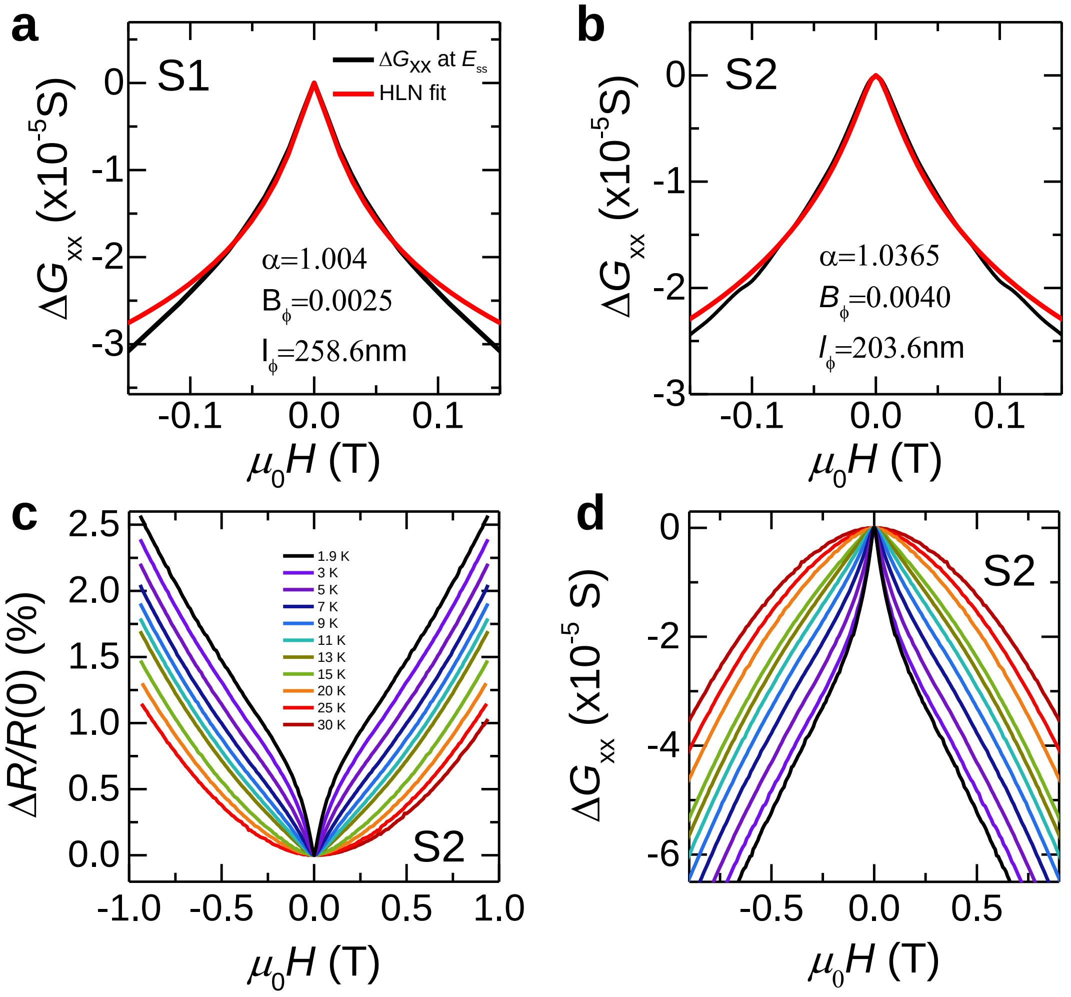

Supplementary Figure 2| The change in the longitudinal conductance ΔG_{xx} and magnetoresistance $\Delta R_{xx}/R_{xx}(H=0)$ in Bi_2Te_3 with E_F within the bulk gap. Fit of ΔG_{xx} to 2D localization theory [19] for two samples: **a**, for sample S1 (121 nm thick) and for **b**, sample S2 (75 nm thick). From the fit we obtain the characteristic phase coherence fields B_ϕ, the dephasing lengths l_ϕ, and the parameter α indicating the number of quantum interference channels. It is evident that in spite of the almost factor of 2 difference in thickness, the fits yield both α and l_ϕ values that are in good correspondence. The decoupling of the surface channels from the bulk, each contributing $\alpha \cong 0.5$ and the similarity of the fits for different thicknesses implies that doping gradient is not a significant factor here. The obtained value of $\alpha \cong 1$ indicates contributions from top and bottom surfaces, each corresponding to one 2D quantum channel with $\alpha = 1/2$, see Refs. [20, 21]. Evolution of the WAL cusp with increasing temperature for sample S2 in **c**, magnetoresistance and **d**, magnetoconductance.

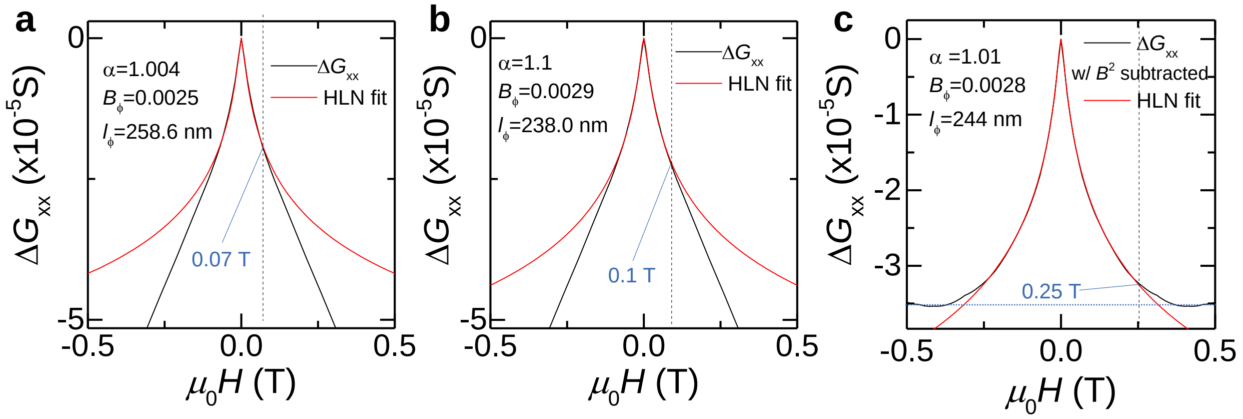

Supplementary Figure 3| 2D localization theory (HLN) fits to WAL. a, The change in the longitudinal magneto-conductance ΔG_{xx} with E_F within the bulk gap (from Supplementary Figure 2a). We used a numerical Monte Carlo technique to fit our transport to the HLN 2D localization theory [19], see text. Minimization of χ^2 in the fit results in the field range of 0.07 T, and slight deviation (crossing) of the data from the fit at low fields. The fit parameters in the fit shown are $\alpha = 1.004$ and $l_\phi = 258.6$ nm. **b**, Enforcing a somewhat higher field fitting range eliminates crossings and gives a slightly larger $\alpha = 1.1$ and a slightly shorter $l_\phi = 238.0$ nm. The reason for the small field range of the fit is a large contribution $\propto B^2$, characteristic of Bi_2Te_3. **c**, Subtracting the B^2 background extends the fitting range to $\sim 0.25T$, which is typical for this system. It is evident that all fits yield value of $\alpha \approx 1$ that are in close correspondence.

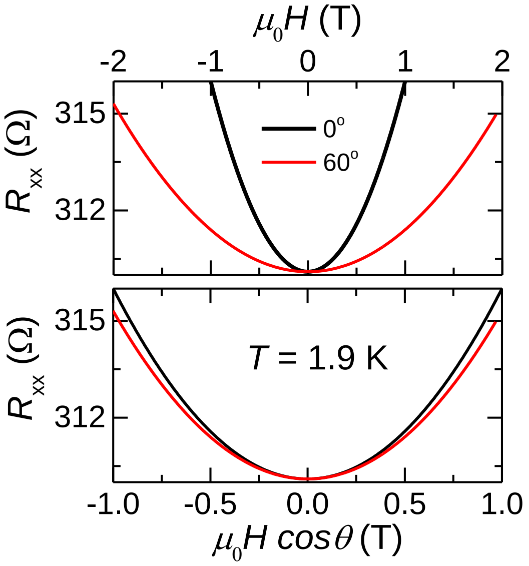

Supplementary Figure 4| Magnetoresistance anisotropy in the B^2 regime at higher fields. In our experiments, in pristine samples without hydrogenation the WAL cusp in Bi_2Te_3 is not detected, as the bulk behavior appears to dominate (see Fig. 2b). The cusp articulates fully when we tune the system's Fermi level E_F upwards past the Dirac point and further away from the bulk valence bands. In the WAL regime, the scaling with $H_\perp = Hcos\theta$ characteristic of 2D transport is observed (Fig. 2d). This scaling is not observed otherwise, as illustrated in the bottom panel. Top panel illustrates that magnetoresistance (MR) in the B^2 regime (no cusp). shown here for two field tilt angles: $\theta = 0$ and $\theta = 60°$, is consistent with the anisotropy of bulk Bi_2Te_3 [22].

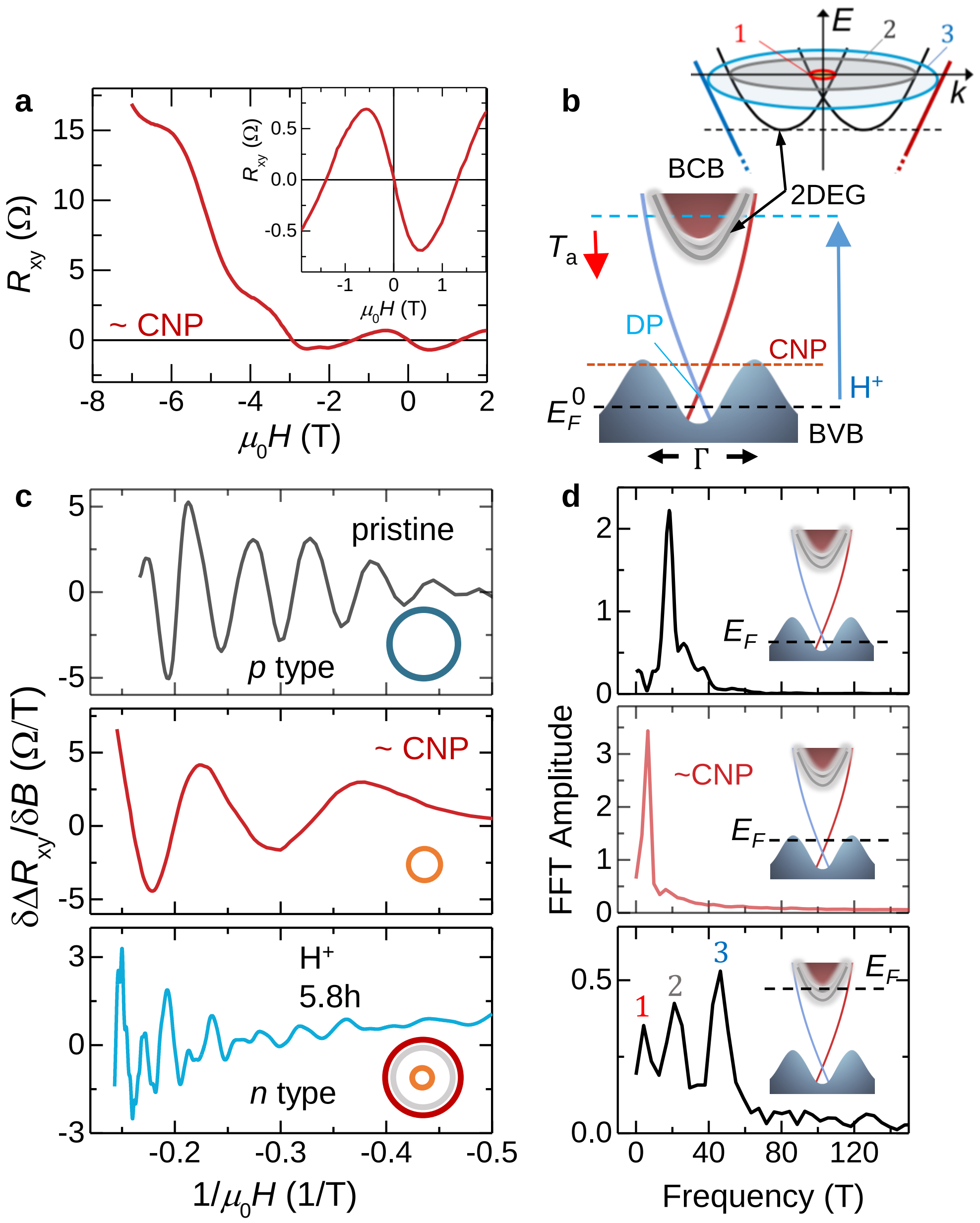

Supplementary Figure 5| Shubnikov de Haas (SdH) quantum oscillations under hydrogenation.a, In the compensated region near the CNP there are both carrier types from the surfaces and the bulk. At higher fields Hall slope is negative and large SdH oscillations are observed, as expected from the majority n-type Dirac surface carriers. Inset: nonlinear Hall resistance near zero field. **b**, Bandstructure cartoon illustrates the upshift of the Fermi level E_F on hydrogenation (cyan arrow) and the reversal by de-hydrogenation (red arrow). In Bi_2Te_3, the E_F^0 at the CNP is slightly above the Dirac point (DP) [23, 24]. Bulk conduction and valence bands are labeled BCB and BVB correspondingly. The 2D subsurface bulk states (2DEG) [25] at the bottom of BCB are shown in grey. 2DEG bands are also present in our DFT calculations (Supplementary Figure 10). A sketch on the top right depicts the relative sizes of the Fermi surface cross-sections for the Dirac (labeled 3) and two Rashba-split 2DEG bands (1 and 2) near the bottom of BCB. They correspond to three periods observed in SdH, shown on the bottom panel of Supplementary Figure 5d. **c**, SdH oscillations of $\partial \Delta R_{xy}/\partial B$ in Bi_2Te_3 with magnetic field applied along the c-axis: (*top*) Before hydrogenation, (*middle*) near the CNP, and (*bottom*) after 5.8 h exposure to H^+. In each case the sizes of Fermi surfaces are illustrated as circles. A single SdH frequency is dominant in the pristine sample and near the CNP. Near the bottom of BCB, the 2DEG states

contribute two additional oscillation periods (two more circles). **d**, The corresponding FFTs: (*top*) In a pristine *p*-type Bi_2Te_3. Insert: a bandstructure cartoon with the initial position of E_F. (*middle*) The peak shifts to lower frequency at $E_F \approx$ CNP. (*bottom*) Three distinct frequencies associated with surface and 2DEG bulk bands appear as E_F nears the bottom of BCB after 5.8 h of hydrogenation. After such longer HCl exposure, as E_F enters BCB the Fermi surface size becomes large again, albeit now with three oscillation periods, see panels **c** and **b**, reflecting the Bi_2Te_3 bandstructure with *n*-type Dirac bands (3) and two 2DEG bands (1 and 2) corresponding to labels in panel **b**. See Table 2 below.

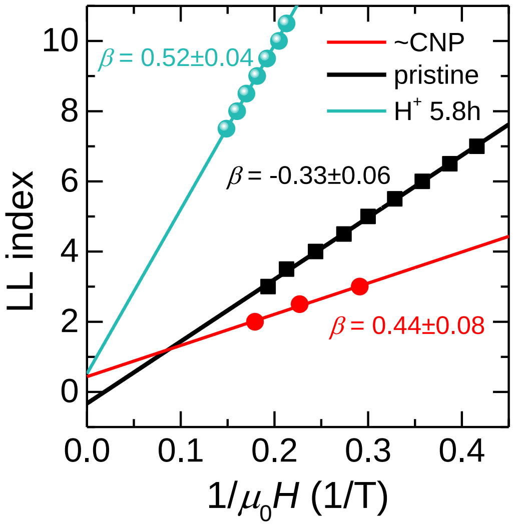

Supplementary Figure 6| Topological π-Berry phase from SdH oscillations in Bi_2Te_3 under hydrogenation. The Landau level (LL) index plot *vs.* field maxima/minima of the SdH oscillations in Supplementary Figure 5c yields an estimate of Berry phase $\varphi_B = 2\pi\beta$. Here, Berry phase was obtained from SdH using a semiclassical description for oscillations in R_{xy} commonly used in 3D TIs [26], $\partial\Delta R_{xy}/\partial B = A_{SdH}\sin[2\pi(\frac{B_F}{B} + \frac{1}{2} + \beta)]$, where A_{SdH} is the oscillation amplitude, B_F is the frequency in $1/B$, and β is the Berry factor. The well-established nontrivial band topology of Bi_2Te_3 [24] should be marked by the appearance of topological Berry phase [23] of π, corresponding to $\beta = 0.5$. It is evident that after hydrogenation for 5.8 hours the Berry factor is $\beta = 0.52 \pm 0.04$, as expected for the topological Dirac particles. Near the CNP $\beta = 0.44 \pm 0.8$ is a within the error bar of 0.5. The error bar is larger here since the oscillation period near the CNP becomes long. This indicates that band topology is not perturbed under hydrogenation. In the pristine sample $\beta = -0.33 \pm 0.06$ is much smaller and negative. There, the contribution of bulk parabolic bands is at play and the phase shift of the quadratic band SdH oscillations depends on the position of the Fermi level in the coexisting linear Dirac band — consequently negative phase shifts can be acquired [27].

We note that, generally, in systems with more complex bandstructures and/or with strong particle correlations π-Berry phase deduced from the LL fan should be taken with some caution. A symmetry analysis of 3D Weyl/Dirac semimetals and certain crystalline and Z_2 topological insulators [28] suggests that a nontrivial band topology is not guaranteed by the LL-fan determined π-Berry phase alone. This has been recently demonstrated [29] in the highly conductive 3D Dirac semimetal Cd_3As_2 and heavy-fermion $LaRhIn_5$, and also for a trivial insulator Bi_2O_2Se, where a different methodology involving a T^2 dependence of the oscillation frequency was employed.

	Frequency (T)	Period (1/T)	S_F ($\times10^{-3}$ /Å^2)	k_F ($\times10^{-2}$ /Å)	n_{2D}^{sdH} ($\times10^{12}$ cm^{-2})	n_{3D}^{sdH} ($\times10^{17}$ cm^{-2})
pristine	18.66	0.05360	1.781	2.381	1.80	9.12
~CNP	*6.548*	*0.1527*	*0.625*	*1.411*	*0.63*	*1.96*
1	4.222	0.2369	0.403	1.133	0.408	1.00
2	21.11	0.04737	2.015	2.533	2.04	11.0
3	46.44	0.02153	4.433	3.757	4.48	35.8

Supplementary Table 2| Fermi cross-sections, Fermi vectors, and carrier densities from SdH oscillations. Fermi cross-sections S_F were obtained from FFT analysis of SdH oscillations in Supplementary Figure 5. The 2D carrier density in pristine Bi_2Te_3, $n_{2D} \cong 2 \times 10^{12}\text{cm}^{-2}$, is much higher that at the CNP, where Fermi cross-section S_F is much smaller and $n_{2D} \cong 6 \times 10^{11}\text{cm}^{-2}$. When the Fermi energy E_F is at the bottom of BCB after 5.8 h of hydrogenation, three S_Fs are resolved (Supplementary Figure 5b); the additional oscillation periods arise from the Rashba-split 2DEG bulk states [25] . The 2D carrier density n_{2D} was calculated from the SdH oscillation period $\Delta(\frac{1}{B}) = \frac{2e}{h \cdot c \cdot n_{2D}}$ (times 2, to account for top and bottom surfaces). The factor of 3 difference between n_{3D} obtained from SdH and Hall arises from the hexagonal warping of the Fermi surface (see Table S1), which is particularly pronounced on the p-side of pristine Bi_2Te_3 [17, 18] and near the CNP. On the n-side of the CNP (labeled 1,2 and 3), $n_{2D} \approx 5 \times 10^{12}$ cm^{-2} is comparable to the one obtained from Hall data, and in line with that reported in high quality thin films [30]. See e.g., also Ref. [31] for comparison.

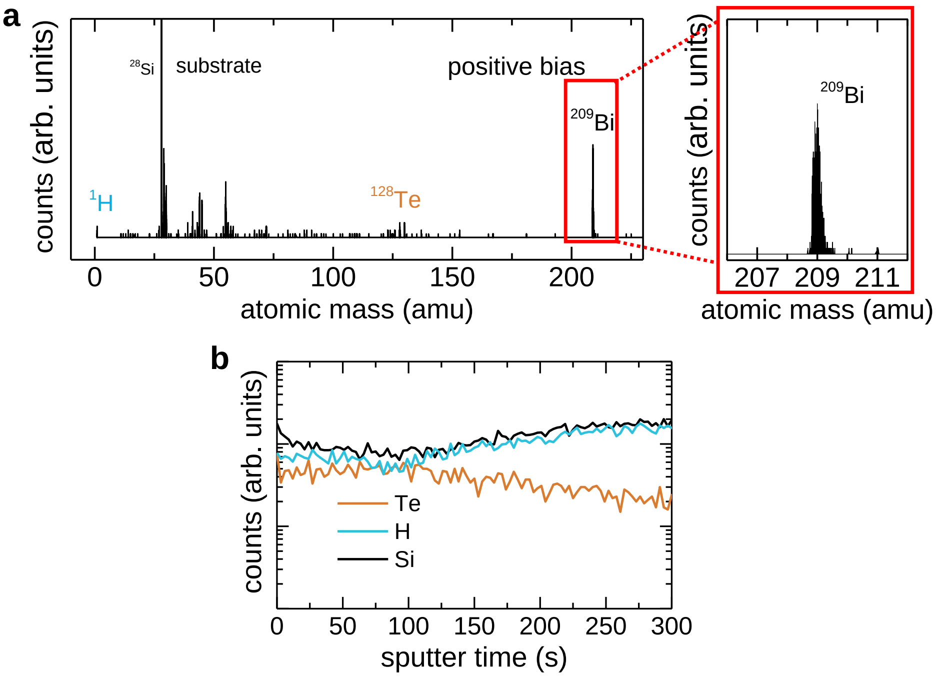

Supplementary Figure 7| ToF-SIMS mass spectrum under positive bias and depth profiling. **a**, *Left*: Under positive bias the mass spectrum shows a strong Bi^{3+} peak and the expectedly suppressed Te and H peaks. *Right*: A zoom of the vicinity of Bi peak shows no additional peaks belonging to a Bi$-$H moiety. **b**, The depth profiling of a small, exfoliated crystal shows that up to

a 300 s sputter time the Te, H, and Si elements are present roughly at the same count level. The Te count is reduced as the sample area is decreased, but hydrogen and Si counts increase somewhat and are similar, indicating that the some of the profiled hydrogen is coming from the substrate. This ease of hydrogen incorporation suggests that it also easily enters our samples.

Mass Number	120	122	123	124	125	126	128	130
Natural Abundance	0.09%	2.55%	0.89%	4.74%	7.07%	18.84%	31.74%	34.08%

Supplementary Table 3| Naturally occurring isotopes of Te. Data adapted from Ref. [32].

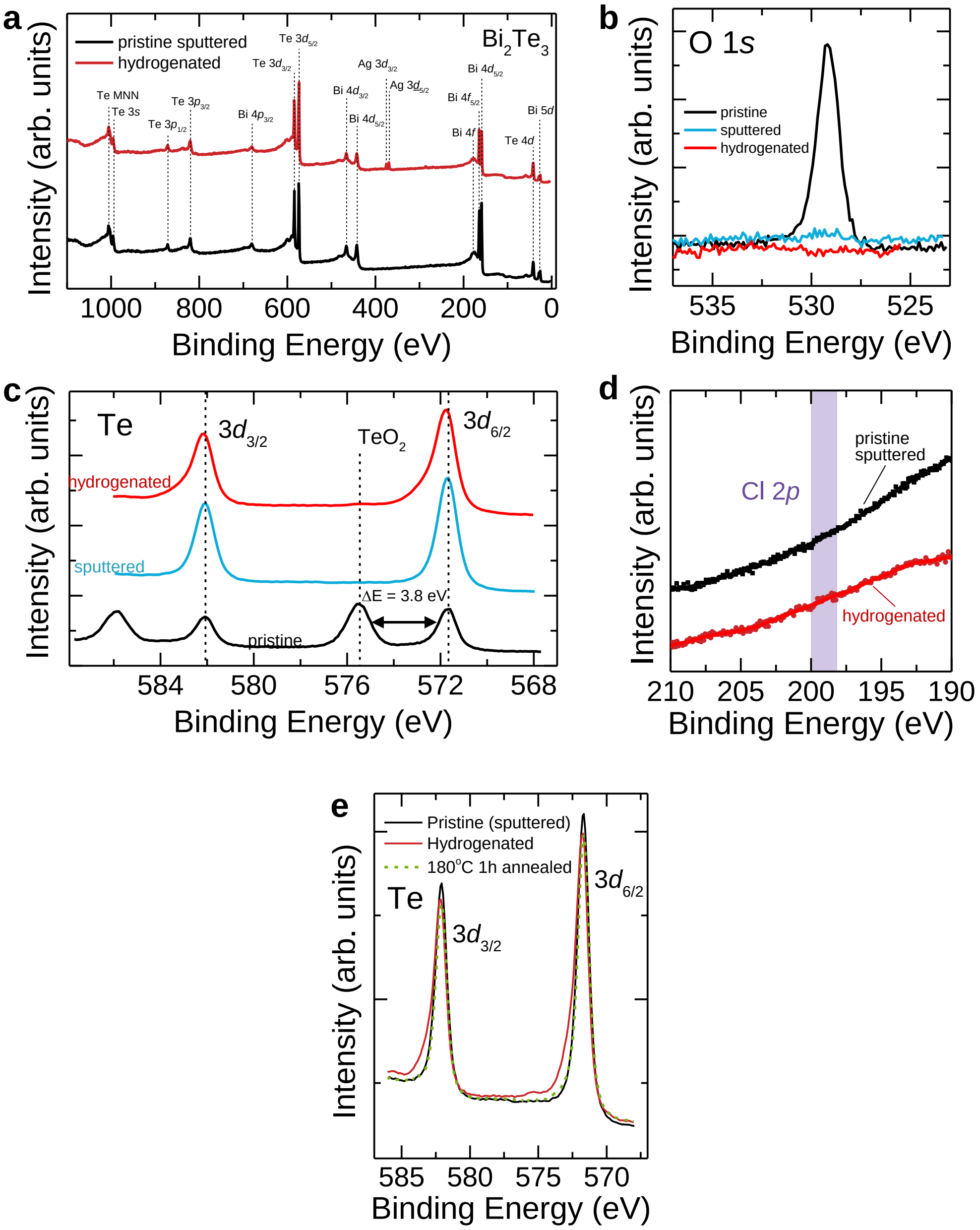

Supplementary Figure 8| X-ray photoelectron spectroscopy (XPS) in pristine and hydrogenated Bi_2Te_3. **a**, All known Bi_2Te_3 XPS peaks are identified in a pristine sample (black), which was Ar-sputtered for 60s to remove native oxide. The same peaks (sans TeO_2) are present

after hydrogenation (red). Small amount of Ag from silver epoxy (used in securing the sample) is detected. **b**, Oxygen O 1s peak (from the surface TeO_2) in a pristine sample is fully removed by hydrogenation. Hydrogenation keeps the surface oxygen-free for at least an hour in air. The process is as efficient as 60s Ar sputtering, as shown for comparison in **b** and **c**. **c**, Native Te oxide peaks are separated by $\Delta E = 3.8$ eV, easily distinguishable from H−Te peaks with $\Delta E = 1$ eV (see Fig. 3g in the main article). **d**, Chlorine (Cl) is not detected (within the 0.1% sensitivity limit) in either pristine (black) or hydrogenated (red) samples — Cl 2p peaks are expected within the purple-shaded range. **e**, Te 3d peaks in pristine, hydrogenated (see analysis in Fig. 3g), and subsequently annealed Bi_2Te_3 show that the hydrogenation process is fully reversible.

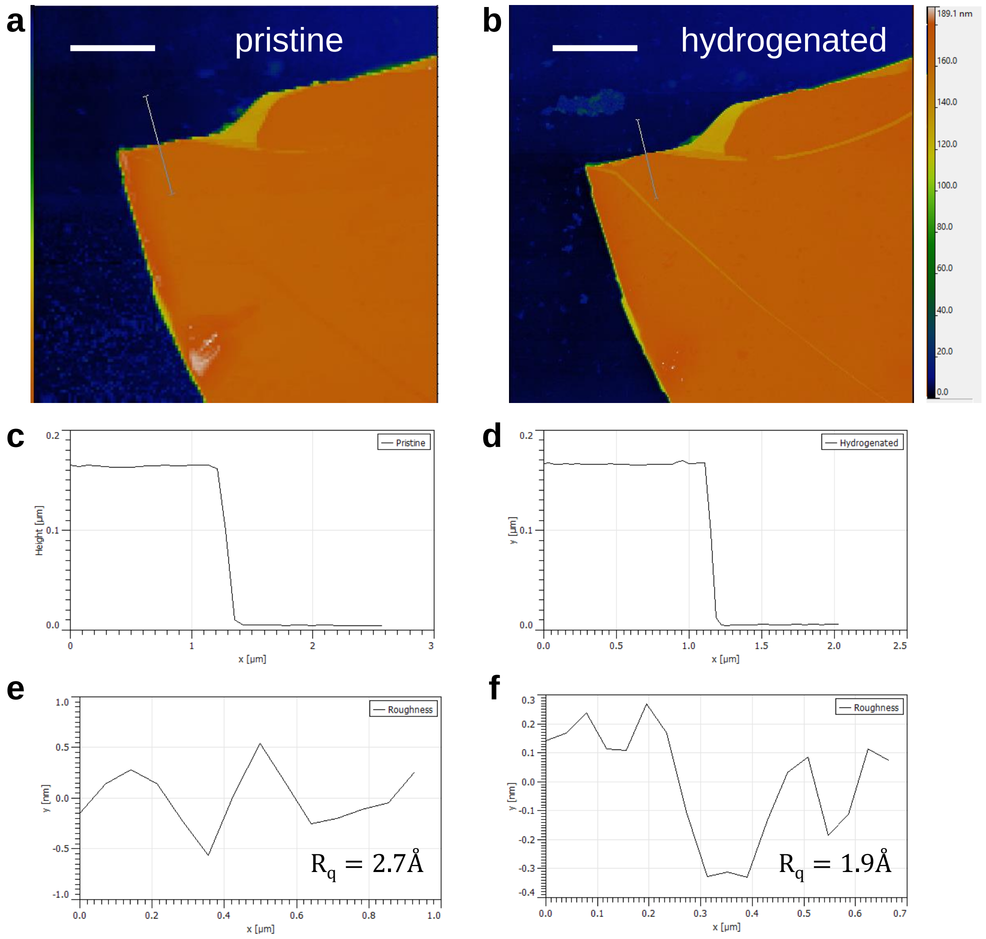

Supplementary Figure 9| Surface morphology of pristine Bi_2Te_3 and after hydrogenation. Atomic force microscopy (AFM) images of a 165 nm thick exfoliated Bi_2Te_3 crystal **a**, before hydrogenation and **b**, after 16 hours of exposure to HCl. The scale bar is 2 μm. HCl removes some of the visible surface contamination on the left of the image in **a** and removes surface oxide, exposing the preexisting cracks in this sample . The surface morphology of the crystal remains intact. **c**,**d**, The corresponding thickness scans along the thin grey lines in both images show that the sample thickness remains unchanged at 165 ± 0.5 nm. The net zero thickness change is because oxide layer is removed by HCl, while the crystal slightly expands along the c-axis (see Fig. S13) — HCl does not etch the TI. **e**,**f**, The surface root-mean-square (RMS) roughness remains at ≈ 0.2 nm, better or comparable to that in epitaxial films grown by MBE [33, 34].

Bi-4f	Te-3d	Cl-2p	O-1s
30.17%	69.83%	0.00%	0.00%

Supplementary Table 4| Percent occurrence of Bi, Te, Cl, and O detected by XPS in hydrogenated Bi_2Te_3. The sample was exposed to air for $\sim$ 1h after hydrogenation. Neither oxygen or chlorine are detected in XPS in hydrogenated Bi_2Te_3.

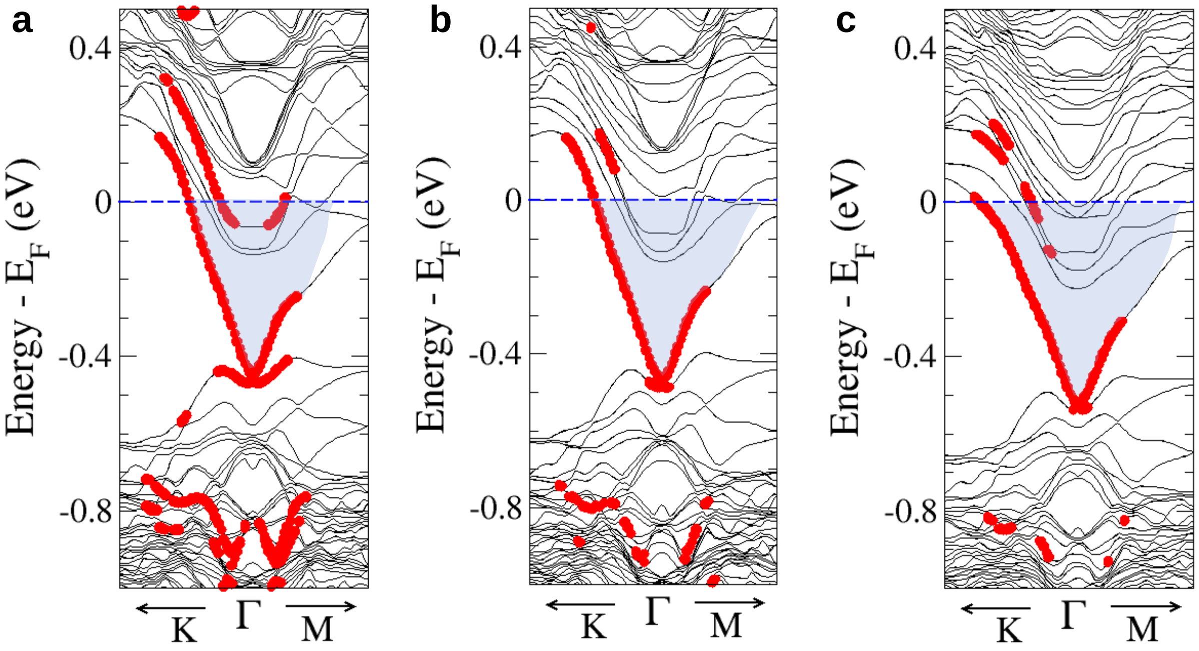

Supplementary Figure 10| DFT-calculated band structures for <u>three</u> cases of H-Te bonding in Bi_2Te_3. **a**, Interstitial, H-Te(2) bonding within QL. **b**, H-Te(1) bonding in the vdW gap and **c**, H-Te(1)-H bonding in the vdW gap (H_2Te). The latter configuration is energetically less favorable. Surface states are denoted as red color. In addition to Dirac surface bands, the two bands with smaller cross-sections, indicated in the DFT calculations in dark blue, are identified as 2DEG subsurface bands. The presence of the 2DEG bands rationalizes our experimentally observed three different Fermi surface cross-sections near the bottom of BCB (manifested by three different periods in SdH oscillations in Fig. S5). Indeed, the ratios of Fermi vectors k_F from SdH relative to the dominant Dirac bands corresponding to peaks labeled 1, 2, and 3 (Dirac) in the bottom panel of Supplementary Figure 5d are 1 : 2 : 3 = 0.302 : 0.674 : 1 (see Table S2). These ratios are in a remarkably good correspondence with the DFT calculations where 1 : 2 : 3 = 0.384 : 0.538 : 1. We note the 2DEG bands are not significantly modified by the three possible cases of H-Te bonding.

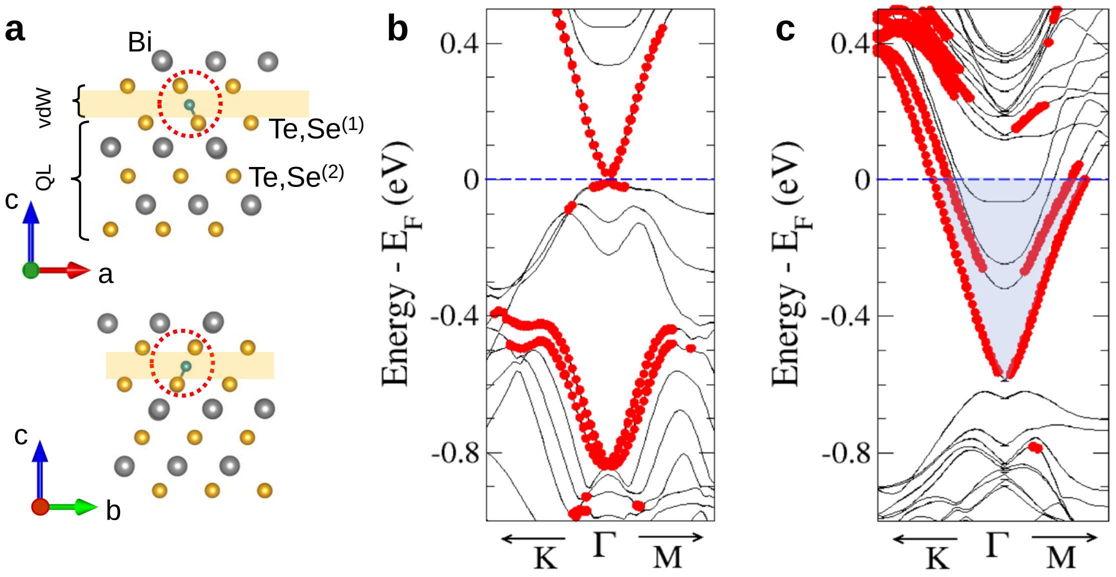

Supplementary Figure 11| DFT-calculated band structures for H:Bi_2Se_3. a, Crystal structure cartoon showing H-Se(1) or H-Te(1) bonding after relaxation. **b**, Pristine 4-QL slab. **c**, H-Se(1) bonding in the vdW gap for the 4-QL slab. The Fermi level is shifted to conduction bands with the introduction of hydrogen. The small gap at Γ point is caused by hybridization between top and bottom surface states due to small slab size in the calculation. For thicker slabs the surface-state gap is closed, which is the case for the samples which were measured. Surface states are denoted as red color. The strong surface hybridization prevents us from identification of the bottom-part of the Dirac cone.

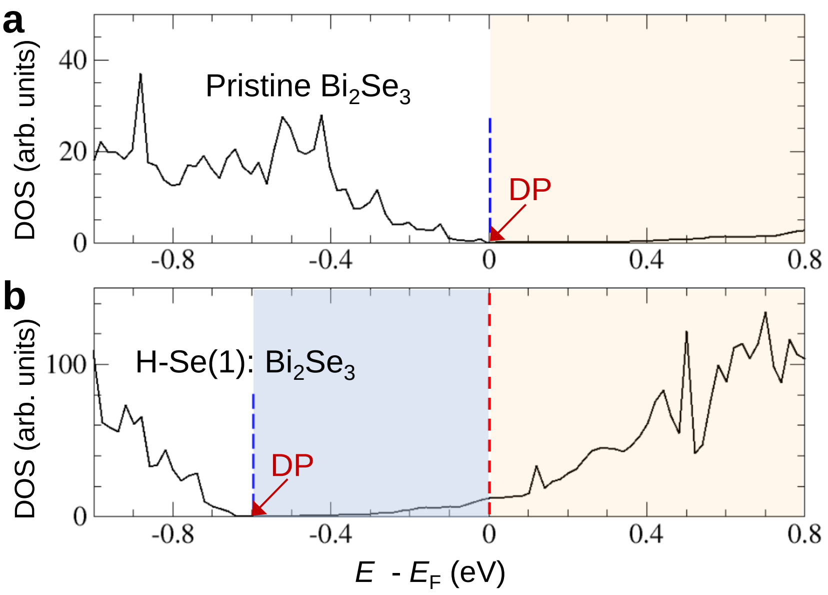

Supplementary Figure 12| DFT-calculated electronic density of states (DOS) for H:Bi_2Se_3. a, Pristine 4-QL slab. **b**, The Fermi level is shifted toward BCB above DP in case of H-Se(1) bonding in the vdW gap for the 4-QL slab. The Dirac point is labeled as DP in both **a** and **b**.

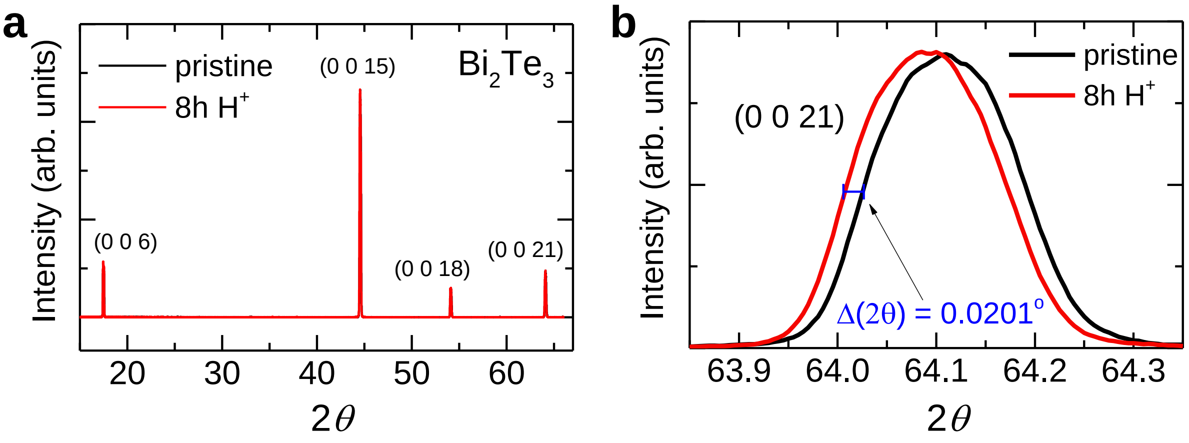

Supplementary Figure 13| Single crystal XRD spectrum of pristine and hydrogenated Bi_2Te_3. a, Hydrogenation leaves crystal structure intact, here shown after 8 hour exposure to HCl. **b**, A detectable elongation of the *c*-axis lattice parameter (≈ 0.00519Å) after hydrogenation is due to uniform expansion along the *c*-axis as hydrogen is incorporated into vdW gaps. The full-width-half-max (FWHM) of the XRD peak is unchanged, indicating absence of hydrogen-induced structural disorder and the associated inhomogeneities.

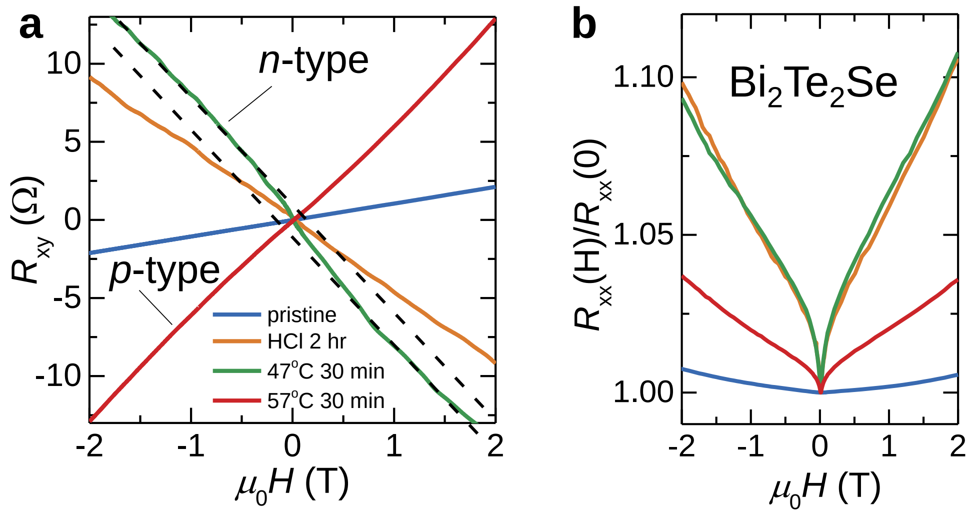

Supplementary Figure 14| Conduction type conversion in Bi_2Te_2Se by hydrogenation. a, Hall resistance R_{xy} *vs.* magnetic field H after hydrogenation and on annealing at different temperatures T_a. The conversion from p- to n-type and back is indicated by the sign change of the slope dR_{xy}/dH. **b**, Evolution of magnetoresistance under annealing implemented to tune Bi_2Te_2Se crystal to stable CNP; it evolves from a quadratic field dependance of a typical bulk metal to a weak antilocalization (WAL) regime with the characteristic low-field cusp near CNP [20, 21].

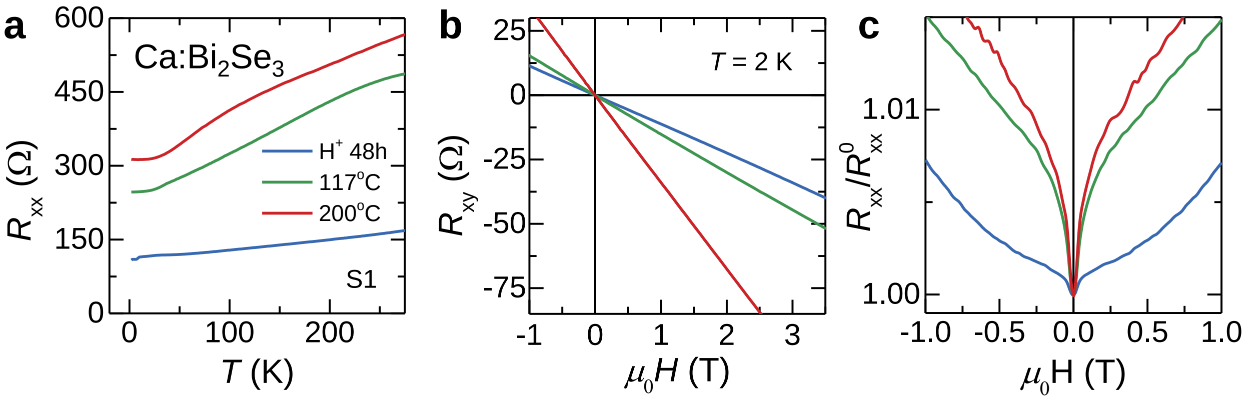

Supplementary Figure 15| Hydrogenation induced *n*-type doping in Ca(0.4%):Bi_2Se_3. a, During the dehydrogenation process of a heavily hydrogenated sample S1 (48 hours), the longitudinal resistance R_{xx} is increased. **b**, Concurrently, the Hall slope becomes steeper as E_F is downshifted towards the initial position (i.e. n-type closer to CNP). **c**, The WAL from SS is more apparent as E_F is downshifted towards the bulk gap.

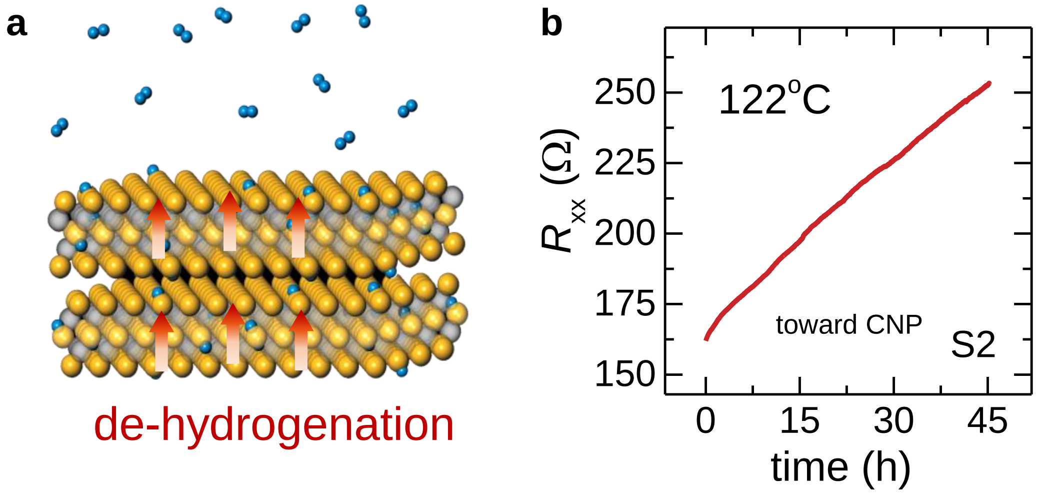

Supplementary Figure 16| In situ monitoring of longitudinal resistance during *in-situ* annealing in a 24h hydrogenated Ca:Bi_2Se_3 device. It allows for a precise control of Fermi level and carrier density.**a**, A cartoon of de-hydrogenation by thermal annealing. **b**, Increase of the longitudinal resistance R_{xx} with annealing time at 122°C toward CNP.

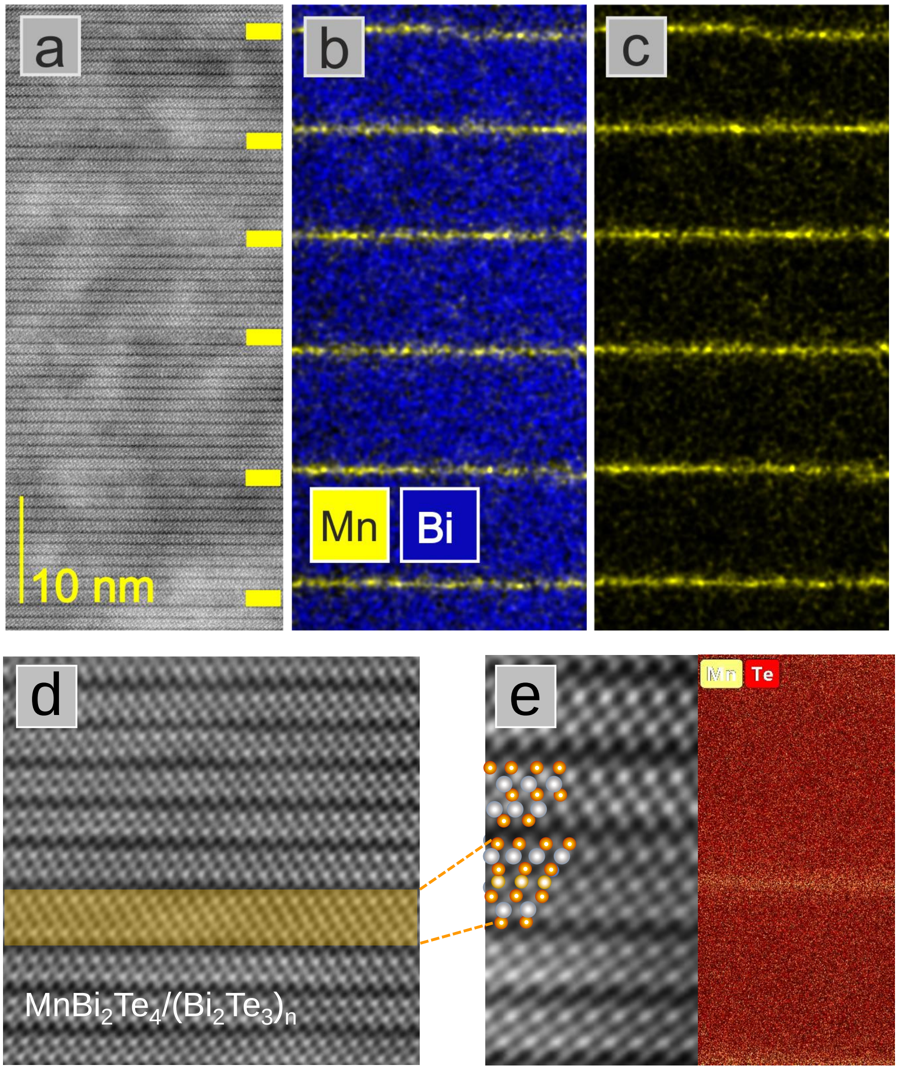

Supplementary Figure 17| HAADF-STEM images of $MnBi_2Te_4/Bi_2Te_3$ superlattice with the corresponding EDX elemental mapping. **a**, Cross-sectional area of an intrinsic $MnBi_2Te_4/Bi_2Te_3$ superlattice consisting of septuple layers (SLs) of $MnBi_2Te_4$ and quintuple layers (QLs) of Bi_2Te_3, with a mostly quasi-periodic (SL-8QL-SL-...) sequence. A larger SL separation of 10-12 QLs is also occasionally observed. The EDX elemental maps in **b**, of Mn and Bi and in **c**, of Mn alone show that Mn is mostly localized in SLs. **d**, HAADF-STEM image of the atomic structure on an expanded scale. Septuple layer (SL) of $MnBi_2Te_4$ SL is highlighted by the yellow shade. **e**, Zoom of the SL region (left) with the corresponding EDX elemental maps of Mn and Te (right).

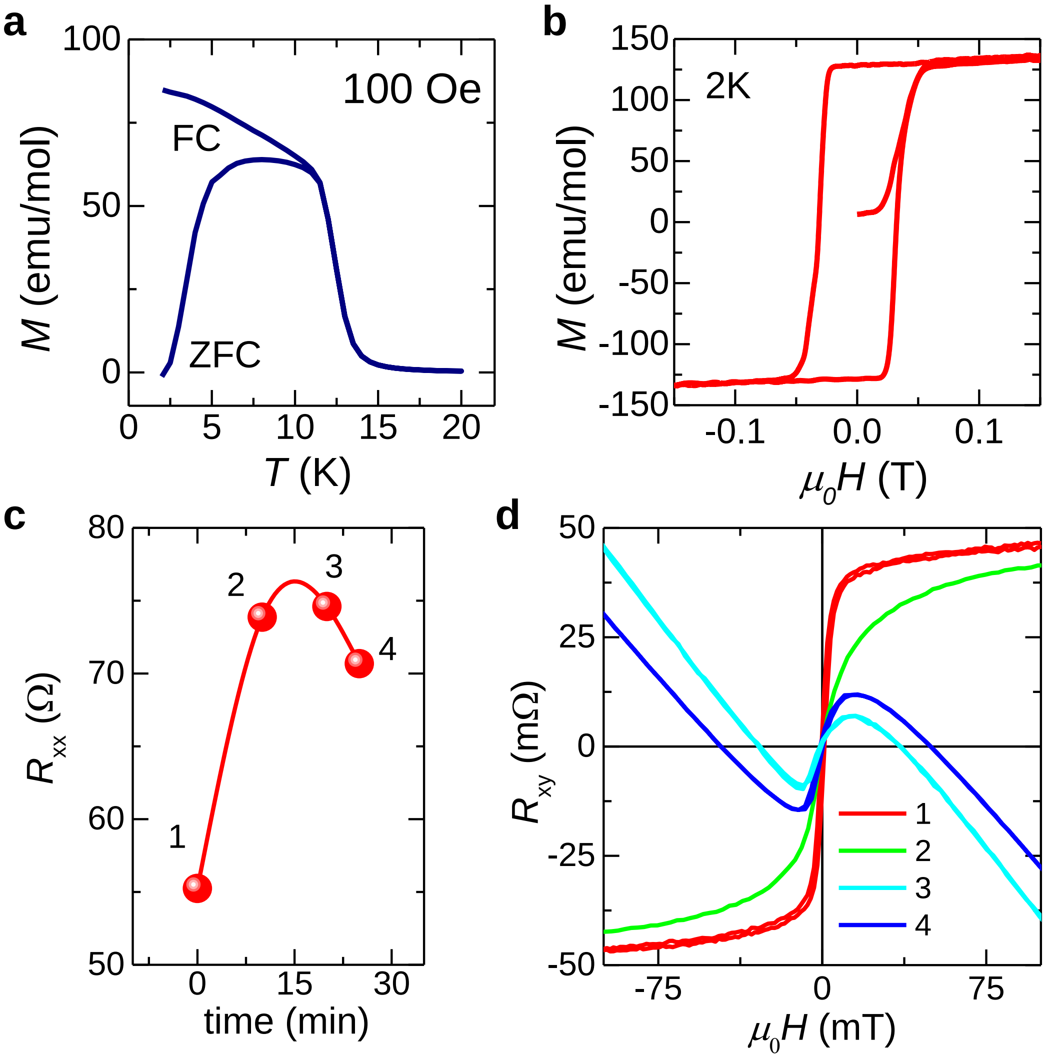

Supplementary Figure 18| Magnetic and electrical transport characterization of a single crystal $MnBi_2Te_4/Bi_2Te_3$ superlattice. **a**, Magnetization M *vs.* temperature after zero-field cooling (ZFC) and field-cooling (FC) in a 100 Oe field. **b**, Magnetic hysteresis loop measured at 2 K. **c**, Longitudinal resistance R_{xx} at 2 K as a function of hydrogenation time. **d**, Hall resistance R_{xy} *vs.* magnetic field for different hydrogenation times shows *p*- to *n*-type conversion in the bulk.

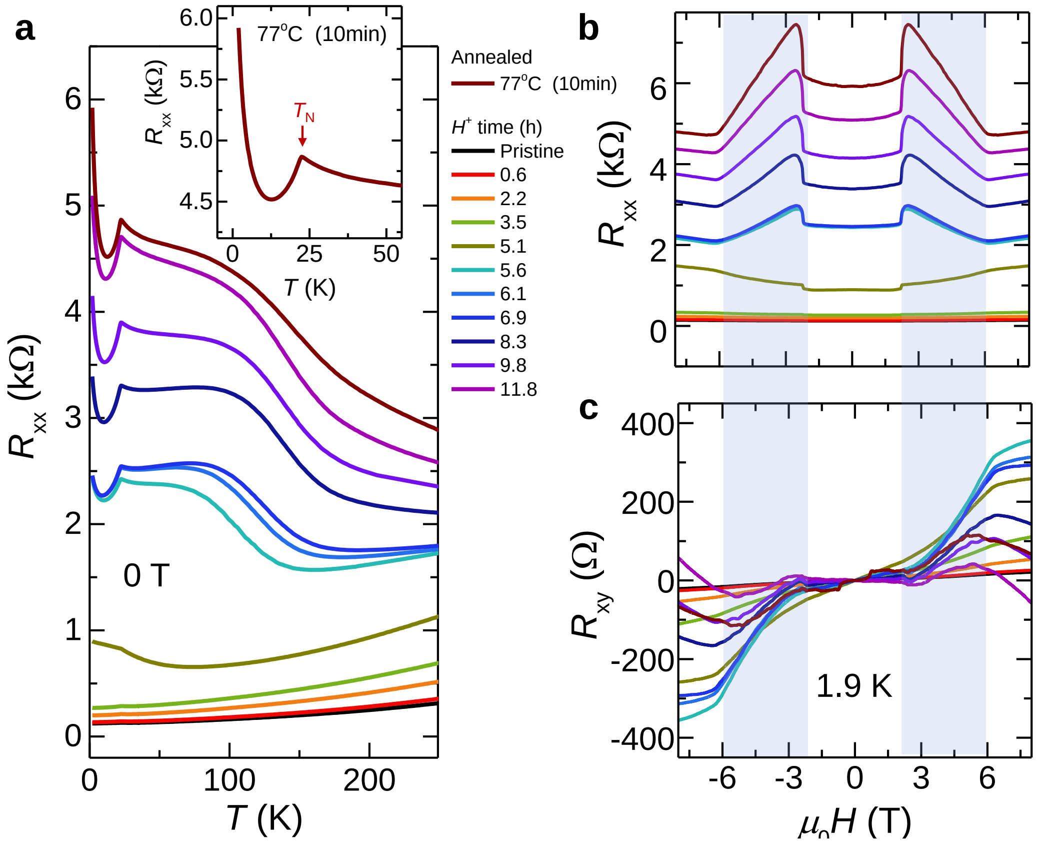

Supplementary Figure 19| Magnetoresistance of Mn($Bi_{0.6}Sb_{0.4}$)$_2$$Te_4$ (MBST) tuned to the vicinity of the CNP by hydrogenation. **a**, Longitudinal resistance $R_{xx}(T)$ of a 80 nm thick MBST crystal at zero magnetic field showing the metallic behavior in a pristine sample and a transition to the insulating behavior under hydrogenation. Inset: An expanded view of $R_{xx}(T)$ at its maximum near the CNP. Since hydrogenation only tunes the Fermi level E_F and does not reconstruct the bandstructure, the antiferromagnetic transition at Néel temperature $T_N \cong 25$ K manifests as a robust hydrogenation-independent cusp. **b**, Longitudinal and **c**, Hall resistances *vs.* magnetic field for different hydrogenation times. Color code is the same in all panels. Hall resistance at 8 T turns from positive to negative after hydrogenating for 11.8 h. The process is reversed by a 10 min thermal anneal at 77°C — Hall resistance changes sign again and the maximum longitudinal resistance of the bulk is achieved (see main Fig. 4e-i). Inserts: Illustration of magnetization alignment stages with external magnetic field. Spins remain antiferromagnetically aligned for $H < 3$ T. The alignment with the applied field proceeds continuously with increasing field in the 3 T $< H <$ 6 T range (indicated as blue shade). The full alignment with magnetic field is obtained for $H > 6$ T. We note that the bandstructure of MBST is eminently tunable from ITM to a Weyl semimetal by Bi-Sb alloying [35]. Ideally, for achieving QAH the magnetic Dirac gap should be in the bulk gap.

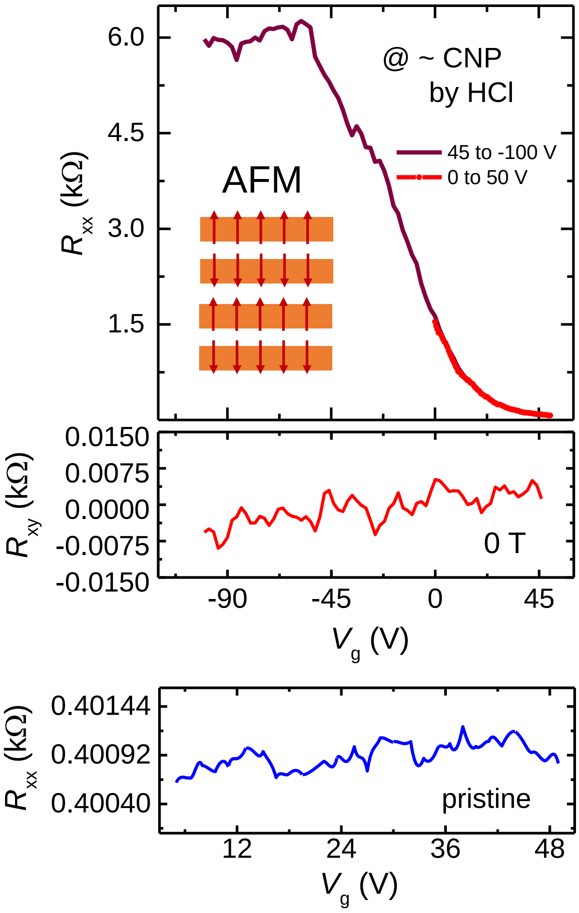

Supplementary Figure 20| Voltage gating of AFM Mn($Bi_{0.6}Sb_{0.4}$)$_2$$Te_4$ (MBST) tuned to the vicinity of the CNP by hydrogenation. *Top:* Electrostatic backgating near the CNP of the hydrogenated ~ 80 nm thick MBST crystal displays a maximum in R_{xx} vs. gate voltage V_g, akin to that obtained for the thick hydrogenated B_2Te_3 (see main Fig. 1). Two voltage sweeps are shown: from 0 to +50 V (red) and from 45 V to -100 V (brown). The asymmetric shape of the gating curve is induced by the bandstructure, as has been seen in other TIs [31]. *Middle:* Hall resistance R_{xy} at zero magnetic field is small and barely changes during the V_g sweep, as expected on a zero-plateau in the axion insulator phase [36]. We note again that the bandstructure of this $Bi_{0.6} - Sb_{0.4}$ AFM system could be possibly near the Weyl semimetal phase and thus requires further study [35]. *Bottom:* The change of $R_{xx}(V_g)$ is nearly null in a pristine MBST crystal, i.e. without hydrogenation the gating effect is negligible.

III. REFERENCES

[1] Van de Walle, C. & Neugebauer, J. Hydrogen in semiconductors. *Annu. Rev. Mater. Res.* **36**, 179–198 (2006).

[2] Pearton, S., Corbett, J. & Borenstein, J. Hydrogen diffusion in crystalline semiconductors. *Physica B* **170**, 85–97 (1991).

[3] Seager, C., Anderson, R. & Brice, D. In situ measurements of hydrogen motion and bonding in silicon. *J. Appl. Phys.* **68**, 3268 (1990).

[4] Tavendale, A., Pearton, S., Williams, A. & Alexiev, D. Injection and drift of a positively charged hydrogen species in *p*-type GaAs. *Appl. Phys. Lett.* **56**, 1457 (1990).

[5] Tavendale, A., Alexiev, D. & Williams, A. Injection and drift of a positively charged hydrogen species in *p*-type GaAs. *Appl. Phys. Lett.* **47**, 316 (1985).

[6] Pankove, J. Photoluminescence recovery in rehydrogenated amorphous silicon. *Appl. Phys. Lett.* **32**, 812 (1978).

[7] Schmidt, J. *et al.* Advances in the surface passivation of silicon solar cells. *Energy Proc.* **15**, 30–39 (2012).

[8] Song, L. *et al.* Laser enhanced hydrogen passivation of silicon wafers. *Intl. J. Photoenergy.* **2015**, 193892 (2015).

[9] Kresse, G. & Furthmüller, J. Efficient iterative schemes for ab initio total-energy calculations using a plane-wave basis set. *Phys. Rev. B* **54**, 11169–11186 (1996).

[10] Kresse, G. & Furthmüller, J. Efficiency of ab-initio total energy calculations for metals and semiconductors using a plane-wave basis set. *Comput. Matls. Sci.* **6**, 15–50 (1996).

[11] Perdew, J., Burke, K. & Ernzerhof, M. Generalized gradient approximation made simple. *Phys. Rev. Lett.* **77**, 3865–3868 (1996).

[12] Blöch, P. Projector augmented-wave method. *Phys. Rev. B* **50**, 17953 (1994).

[13] Kresse, G. & Joubert, D. From ultrasoft pseudopotentials to the projector augmented-wave method. *Phys. Rev. B* **59**, 1758–1775 (1999).

[14] Grimme, S., Antony, J., Ehrlich, S. & Krieg, H. A consistent and accurate ab initio parametrization of density functional dispersion correction (DFT-D) for the 94 elements H-Pu. *J. Chem. Phys.* **132**, 154104 (2010).

[15] Loehlin, J., Mennitt & Waugh, J. Proton resonance study of molecular motion and phase behavior of solid H_2S and H_2Se. *J. Chem. Phys.* **44**, 3912–3917 (1966).

[16] Lide, D. *CRC Handbook of Chemistry and Physics, 87th ed.* (CRC Press, Boca Raton, FL, 2006).

[17] Fu, L. Hexagonal warping effects in the surface states of the topological insulator Bi_2Te_3. *Phys. Rev. Lett.* **103**, 266801 (2009).

[18] Chen, M. *et al.* Hexagonal warping effects in the surface states of the topological insulator Bi_2Te_3. *Sci. Adv.* **5**, eaaw3988 (2019).

[19] Hikami, S., Larkin, A. & Nagaoka, Y. Spin-orbit interaction and magnetoresistance in the two-dimensional random system. *Progress Theor. Phys.* **63**, 707–710 (1980).

[20] Zhao, L. *et al.* Stable topological insulators achieved using high energy electron beams. *Nature*

Comm. **7**, 10957 (2016).

[21] Garate, I. & Glazman, L. Weak localization and antilocalization in topological insulator thin films with coherent bulk-surface coupling. *Phys. Rev. B* **86**, 035422 (2012).

[22] Yue, Z., Wang, X. & Dou, S. Angular-dependences of giant in-plane and interlayer magnetoresistances in Bi_2Te_3 bulk single crystals. *Appl. Phys. Lett.* **101**, 152107 (2012).

[23] Qi, X.-L. & Zhang, S.-C. Topological insulators and superconductors. *Rev. Mod. Phys.* **83**, 1057–1110 (2011).

[24] Zhang, H. *et al.* Topological insulators in Bi_2Se_3, Bi_2Te_3, and Sb_2Te_3 with a single Dirac cone on the surface. *Nature Phys.* **5**, 438–442 (2009).

[25] Bahramy, M. S. *et al.* Emergent quantum confinement at topological insulator surfaces. *Nat. Commun.* **3**, 1159 (2012).

[26] Ren, Z., Taskin, A., Sasaki, S., Segawa, K. & Ando, Y. Large bulk resistivity and surface quantum oscillations in the topological insulator Bi_2Te_2Se. *Phys. Rev. B.* **82**, 241306(R) (2010).

[27] Datta, B. *et al.* Nontrivial quantum oscillation geometric phase shift in a trivial band. *Sci. Adv.* **5**, eaax6550 (2019).

[28] Alexandradinata, A., Wang, C., Duan, W. & Glazman, L. Revealing the topology of Fermi-surface wave functions from magnetic quantum oscillations. *Phys. Rev. X* **8**, 011027 (2018).

[29] Guo, C. *et al.* Temperature dependence of quantum oscillations from non-parabolic dispersions. *Nature Comm.* **12**, 6213 (2018).

[30] Hoefer, K. *et al.* Intrinsic conduction through topological surface states of insulating Bi_2Te_3 epitaxial thin films. *Proc. Natl. Acad. Sci. (USA)* **111**, 14979–14984 (2014).

[31] Lee, J., Park, J., Lee, J.-H., Kim, J. & Lee, H.-J. Gate-tuned differentiation of surface-conducting states in $Bi_{1.5}Sb_{0.5}Te_{1.7}Se_{1.3}$ topological-insulator thin crystals. *Phys. Rev. B.* **86**, 245321 (2012).

[32] Firestone, R., Baglin, C. & Chu, S. *Table of Isotopes, 8th ed. update* (Springer, Hungary, 1998).

[33] Chen, Z. *et al.* Robust topological interfaces and charge transfer in epitaxial Bi_2Se_3/II-VI semiconductor superlattices. *Nano Lett.* **15**, 6365 (2015).

[34] Levy, I. *et al.* Designer topological insulator with enhanced gap and suppressed bulk conduction in Bi_2Se_3/Sb_2Te_3 ultrashort-period superlattices. *Nano Lett.* **20**, 3420–3426 (2020).

[35] Chen, B. *et al.* Intrinsic magnetic topological insulator phases in the Sb doped $MnBi_2Te_4$ bulks and thin flakes. *Nature Comms.* **10**, 4469 (2019).

[36] Liu, C. *et al.* Robust axion insulator and Chern insulator phases in a two-dimensional antiferromagnetic topological insulator. *Nature Mater.* **19**, 522–527 (2020).
